# Genome-wide comparison of the transcriptomes of highly enriched normal and chronic myeloid leukemia stem and progenitor cell populations

**DOI:** 10.18632/oncotarget.990

**Published:** 2013-05-06

**Authors:** Jonathan M. Gerber, Jessica L. Gucwa, David Esopi, Meltem Gurel, Michael C. Haffner, Milada Vala, William G. Nelson, Richard J. Jones, Srinivasan Yegnasubramanian

**Affiliations:** ^1^ The Sidney Kimmel Comprehensive Cancer Center, Johns Hopkins University, School of Medicine, Baltimore, MD, USA

**Keywords:** chronic myeloid leukemia, CML, leukemic stem cell, LSC, normal hematopoietic stem cell, HSC, myeloid progenitor cells, CD34, CD38, ALDH, IL2RA, CD25, DPP4, CD26, GAS2

## Abstract

The persistence leukemia stem cells (LSCs) in chronic myeloid leukemia (CML) despite tyrosine kinase inhibition (TKI) may explain relapse after TKI withdrawal. Here we performed genome-wide transcriptome analysis of highly refined CML and normal stem and progenitor cell populations to identify novel targets for the eradication of CML LSCs using exon microarrays. We identified 97 genes that were differentially expressed in CML versus normal stem and progenitor cells. These included cell surface genes significantly upregulated in CML LSCs: *DPP4* (CD26), *IL2RA* (CD25), *PTPRD*, *CACNA1D*, *IL1RAP*, *SLC4A4*, and *KCNK5*. Further analyses of the LSCs revealed dysregulation of normal cellular processes, evidenced by alternative splicing of genes in key cancer signaling pathways such as p53 signaling (e.g. *PERP*, *CDKN1A*), kinase binding (e.g. *DUSP12*, *MARCKS*), and cell proliferation (*MYCN*, *TIMELESS*); downregulation of pro-differentiation and TGF-β/BMP signaling pathways; upregulation of oxidative metabolism and DNA repair pathways; and activation of inflammatory cytokines, including *CCL2*, and multiple oncogenes (e.g., *CCND1*). These data represent an important resource for understanding the molecular changes in CML LSCs, which may be exploited to develop novel therapies for eradication these cells and achieve cure.

## INTRODUCTION

Despite the significant improvement in survival rates of chronic phase (CP) chronic myeloid leukemia (CML) patients made possible by tyrosine kinase inhibitor (TKI) therapy, cures outside of allogeneic blood or marrow transplantation are rare[1-4]. This appears to be due to the resistance of leukemia stem cells (LSCs) in CML to the pro-apoptotic effects of TKI agents[5-8]. Accordingly, most CML patients who discontinue TKIs while in molecular remission eventually relapse[9]. Moreover, for most of the TKI-induced cytogenetic remissions that remain durable at least 7 years, CML LSCs in these patients can still acquire additions mutations with progression to blast crisis (BC)[10]. Thus, there remains a clear need to identify novel molecular targets specific to the CML LSCs[11].

The precise mechanisms of CML LSC resistance to TKIs are not fully defined. CML LSCs appear to share many biological properties with their normal counterparts[6, 12] that probably limit the effectiveness of therapeutic strategies targeting BCR-ABL signaling. Hematopoietic stem cells (HSCs) are largely quiescent and normally express high levels of the multidrug resistance-1 gene[13], two factors that may limit the cellular uptake of imatinib[14]. Moreover, BCR-ABL expression appears to be required for the survival of CML progenitors but not CML LSCs, where the *BCR-ABL* gene can be silent likely because HSCs already are long-lived and self-renew[12, 15].

Biologic studies on LSCs have been hampered by the relative rarity of these cells, as well as the lack of a consensus on their exact phenotype. LSCs are often phenotypically defined as simply the CD34^+^ leukemia cells or, more recently, the more enriched CD34^+^CD38^−^ subset, but even the CD34^+^CD38^−^ cells are a heterogeneous population of which the LSCs constitute only a fraction[12, 16]. Normal CD34^+^CD38^−^ cells can be further refined for HSCs based on low side scatter and high aldehyde dehydrogenase (ALDH) 1 activity[17, 18]. As few as 1,000 normal CD34^+^CD38^−^ALDH^high^ cells will reproducibly engraft NOD/SCID-IL2R^null^ (NSG) mice[18]. The major biologic function of the ALDH1 family, also known as the retinaldehyde dehydrogenases, is the biosynthesis of retinoic acid, but they also participate in the detoxification of a variety of compounds such as ethanol and active metabolites of cyclophosphamide[19]. We previously reported that high ALDH expression also can distinguish CML cells capable of engrafting NSG mice (i.e. CML LSCs) from more differentiated CML progenitors within the CML CD34^+^CD38^−^ population[20]. Importantly, expression of putative therapeutic targets by CML progenitor cells was not necessarily representative of that in the CML LSCs[20], highlighting the need to search for new targets in refined LSC populations. Here, we report a comprehensive transcriptional profile of CML LSCs as compared to normal HSCs and identify unique cell surface molecules and mechanistic pathways that may serve as potential CML LSC targets.

## RESULTS

### Identification of potential targets that can distinguish CML LSCs from normal HSCs

**Figure 1:**
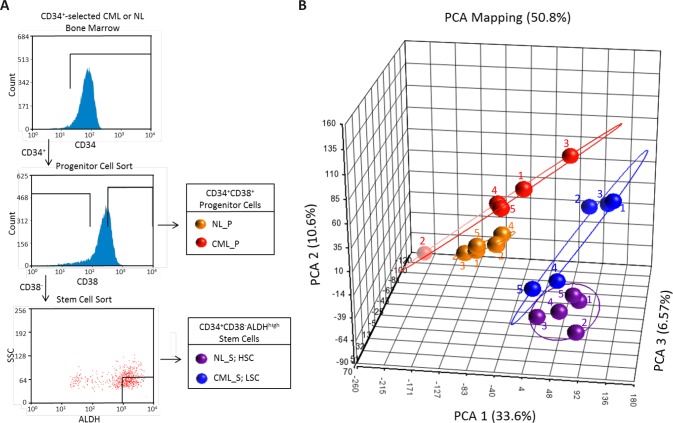
Global gene expression patterns in CML and normal stem and progenitor populations (A) Cell sorting schematic for isolation of stem CD34^+^CD38^−^ALDH^high^ and CD34^+^CD38^+^ cells. A representative CML sample is shown. An analogous strategy was used to sort normal (NL) samples. (B) Principal components analyses (PCA) were done on microarray gene-level expression data for CML and normal CD34^+^CD38^−^ALDH^high^ and CD34^+^CD38^+^ cell populations. CML_S (blue symbols), chronic myeloid leukemic stem (CD34^+^CD38^−^ALDH^high^) cells; CML_P (red symbols), chronic myeloid leukemic progenitor (CD34^+^CD38^+^) cells; NL_S (purple symbols), normal stem (CD34^+^CD38^−^ALDH^high^) cells; NL_P (yellow symbols), normal progenitor (CD34^+^CD38^+^) cells. Sample IDs correspond to Supplementary Table 1.

In order to characterize the expression profile of CP CML LSCs and identify potential therapeutic targets unique to this population, we sorted CD34^+^CD38^+^ and CD34^+^CD38^−^ALDH^high^ cells to obtain highly enriched populations of progenitor and stem cells, respectively, from bone marrow of both healthy donors and CP CML patients (Figure 1A; Supplementary Table 1). As already discussed, HSCs are enriched in the CD34^+^CD38^−^ALDH^high^ cells[17, 18], and these cells contain few of the more differentiated colony-forming unit or progenitor cells, which are enriched in the CD34^+^CD38^+^ cell fraction[26]. Likewise, CD34^+^CD38^−^ALDH^high^ cells show enrichment for CML LSCs with enhanced engraftment capabilities in immune deficient mice compared to the remaining CD34^+^CD38^−^ cells[20]. Whole transcriptome profiling of each population was carried out by microarray analysis using an Affymetrix Human Exon 1.0 ST array, allowing measurement of differential gene expression and analysis of alternative transcripts. Principal components analysis of the gene-level data revealed distinct clustering of the four populations and showed that global gene expression patterns between the normal and CML CD34^+^CD38^−^ALDH^high^ cells are closer to each other than normal are to their matched CD34^+^CD38^+^ cells (Figure 1B). Furthermore, the CML subset displayed greater variability in the gene expression patterns than their normal counterparts. Part of this variability in the CML CD34^+^CD38^−^ALDH^high^ fraction could be accounted for by the presence of residual *BCR-ABL* negative normal HSC in this cell population; the two subjects with the highest fraction of residual normal HSC clustered most closely with the normal HSC (Figure 1; Supplementary Table 1).

**Figure 2:**
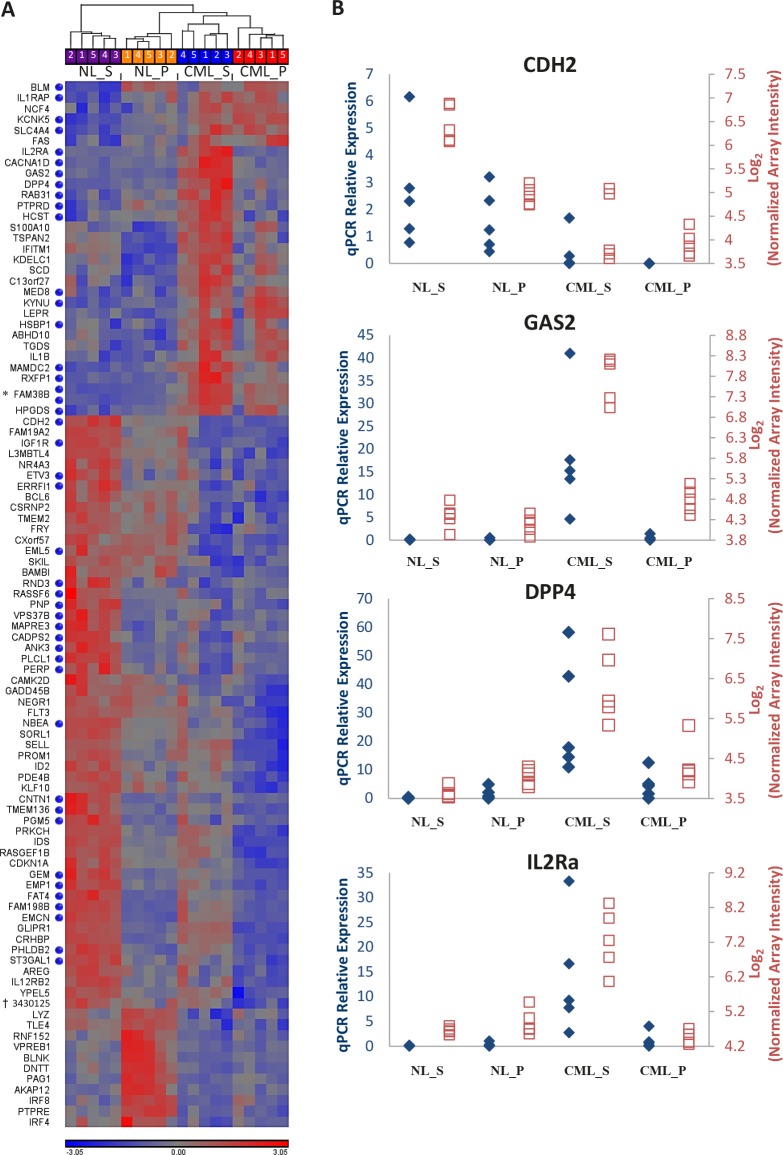
Differentially expressed genes between CML and normal stem and progenitor cells CML_S, chronic myeloid leukemic stem (CD34^+^CD38^−^ALDH^high^) cells; CML_P, chronic myeloid leukemic progenitor (CD34^+^CD38^+^) cells; NL_S, normal stem (CD34^+^CD38^−^ALDH^high^) cells; NL_P, normal progenitor (CD34^+^CD38^+^) cells. A) Heatmap showing expression patterns of genes found by ANOVA to be differentially expressed between CML and normal CD34^+^CD38^−^ALDH^high^ and CD34^+^CD38^+^ cells. Sample IDs correspond to Supplementary Table 1. Blue dots (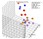) represent genes differentially expressed in CML versus normal CD34^+^CD38^−^ALDH^high^ cells with FDR = 0.05 and |log_2_(Fold Change)| > 1. Upregulated and downregulated expression levels are indicated in red and blue, respectively. *FAM38B is represented by 2 separate Affymetrix transcript IDs (3798778; 3798829). †No gene name associated with Affymetrix transcript ID 3430125. B) Four candidate differentially expressed genes are shown. cDNA was prepared for each sample as described in Methods. To visualize quantitative RT-PCR (qPCR) results (blue axes labels,♦), the relative amount of the gene of interest was determined using the ΔΔ*C*_t_ method. Microarray expression was plotted using log_2_ transformed, default RMA background corrected array intensities (red axes labels,□).

Although global gene expression patterns in the CML and normal CD34^+^CD38^−^ALDH^high^ cells were fairly similar, gene-level analysis allowed us to identify several genes with significant differential expression that may serve as therapeutic targets. Using ANOVA, we identified genes that were significantly differentially expressed between all CML vs. normal samples, regardless of sorted population, and also those that were significantly differentially expressed specifically between CD34^+^CD38^−^ALDH^high^ cell populations of CML and normal samples (FDR = 0.05, |log_2_(Fold Change)| > 1). A total of 97 genes were identified through this analysis and a heatmap was created showing the expression patterns of each gene across the four cell populations (Figure 2A). Notably, expression of this gene set was able to distinguish CML stem and progenitor cells from their normal counterparts by hierarchical clustering. Thirty-one transcripts were found to be upregulated in CML CD34^+^CD38^−^ALDH^high^ cells compared to normal CD34^+^CD38^−^ALDH^high^ or CD34^+^CD38^+^ cells (Figure 2A), representing selective putative CML stem cell targets. These included *BLM*, *FAS*, *KYNU*, *NCF4*, *PTPRD*, *RAB31*, *SCD*, *ABHD10*, and *HPGDS*, genes known to be involved in key cell signaling and metabolic pathways. The most upregulated gene selectively expressed on CML CD34^+^CD38^−^ALDH^high^ when compared to their normal counterparts was *GAS2* (p = 5.96 × 10^−11^, average fold change = 23.5; Figure 2B). To further analyze our list of potential LSC-specific targets, functional annotation by the Database for Annotation, Visualization, and Integrated Discovery (DAVID) of genes differentially expressed on the CML versus normal CD34^+^CD38^−^ALDH^high^ cells (FDR = 0.05, |log_2_(Fold Change)| > 1); represented by (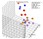) in Figure 2) was carried out and highlighted several plasma membrane-associated genes (GO:0044459, Plasma Membrane Part), including the most up- and down-regulated genes, *DPP4* and *CDH2*, respectively (Table 1). From this list, *DPP4*, *IL2RA*, *RAB31*, *PTPRD*, *CACNA1D*, *IL1RAP*, *SLC4A4*, and *KCNK5* were upregulated in the CML CD34^+^CD38^−^ALDH^high^ population and exhibit a cell surface protein localization. Microarray expression levels were verified by quantitative RT-PCR for a few select interesting genes (Figure 2B). Microarray intensity values were highly correlated with relative expression levels determined by quantitative RT-PCR analysis.

**Table 1:** Plasma Membrane-Associated Genes that are Differentially Expressed in CML versus Normal Stem Cells

Gene Symbol	Fold Change Expression*	P	Genomic Location†	Gene Name
DPP4	9.77	1.23E-06	2q24.3	dipeptidyl-peptidase 4
IL2RA	6.08	3.27E-07	10p15-p14	interleukin 2 receptor, alpha
RAB31	5.13	7.92E-06	18p11.3	RAB31, member RAS oncogene family
PTPRD	5.01	5.02E-06	9p23-p24.3	protein tyrosine phosphatase, receptor type, D
CACNA1D	3.53	8.39E-07	3p14.3	calcium channel, voltage-dependent, L type, alpha 1D subunit
IL1RAP	2.90	7.69E-05	3q28	interleukin 1 receptor accessory protein
SLC4A4	2.50	6.28E-05	4q21	solute carrier family 4, sodium bicarbonate cotransporter, member 4
KCNK5	2.06	5.58E-05	6p21	potassium channel, subfamily K, member 5
CADPS2	-2.29	2.74E-05	7q31.3	Ca++-dependent secretion activator 2
GEM	-2.52	1.58E-05	8q13-q21	GTP binding protein overexpressed in skeletal muscle
ANK3	-2.87	1.35E-05	10q21	ankyrin 3, node of Ranvier (ankyrin G)
PGM5	-2.96	1.13E-04	9q13	phosphoglucomutase 5
IGF1R	-2.96	1.28E-05	15q26.3	insulin-like growth factor 1 receptor
EMCN	-3.12	4.91E-05	4q24	endomucin
CNTN1	-4.32	2.28E-05	12q11-q12	contactin 1
PERP	-4.41	1.31E-04	6q24	PERP, TP53 apoptosis effector
CDH2	-4.73	5.75E-07	18q11.2	cadherin 2, type 1, N-cadherin (neuronal)

Functional annotation results by DAVID are represented in the table, showing genes enriched for gene ontology term “Plasma Membrane Part” (GO:0044459).

* Calculated between CML and normal stem (CD34+CD38-ALDH^high^) cell populations from five CML or normal marrow donors from log_2_ transformed, default RMA background corrected array intensities. Positive values (red) indicate upregulation of gene in CML compared to normal, and negative values (blue) indicate downregulation in CML.

† Genomic coordinates refer to the human reference genome hg19 (GRCh37).

### Dysregulation of proliferation, differentiation and molecular pathways in CML LSCs

**Figure 3:**
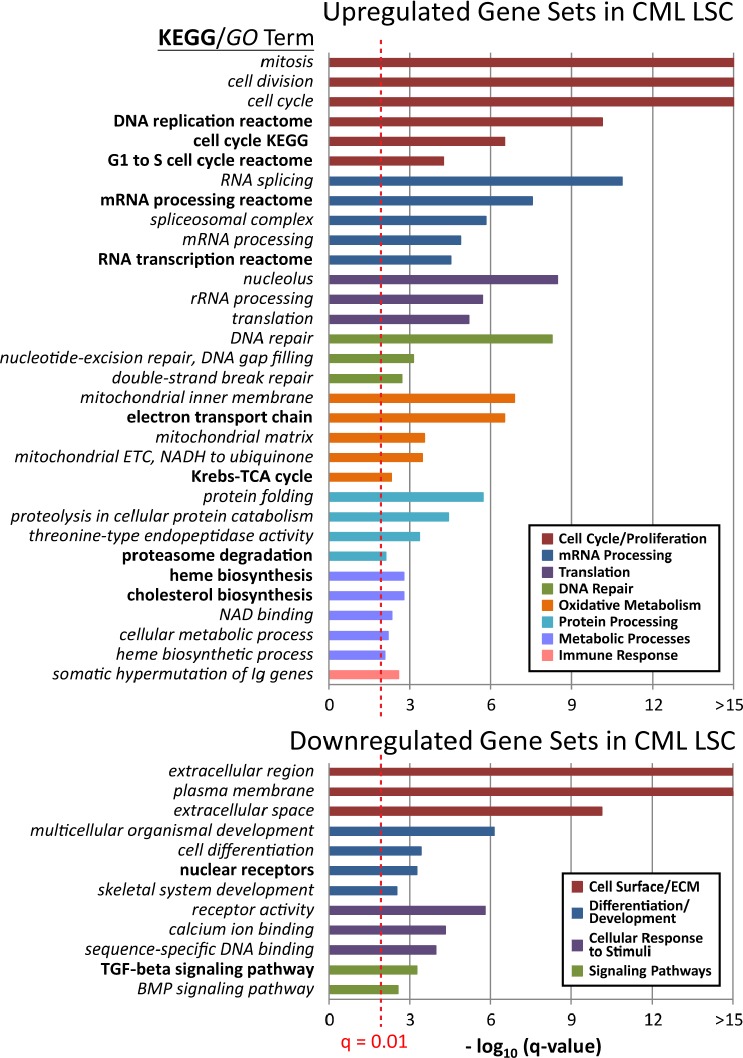
Altered cellular functions and pathways in CML LSCs compared to normal HSCs Gene-set enrichment analyses (GSEA) were carried out to identify upregulated or downregulated GO and KEGG terms in CML versus normal CD34^+^CD38^−^ALDH^high^ cells. Upregulated or downregulated GO and KEGG terms were categorized by common cellular function among a group of associated terms, indicated by bar color. Gene sets with a q-value < 0.01 (red dotted line) were considered significant. Q-value represents the false discovery rate of the p-value, as previously described.[50] Top three GO or KEGG terms in each category are shown. Bold text indicates KEGG terms. *Italicized* text indicates GO terms.

Characterization of the molecular mechanisms underlying malignant transformation of the normal HSCs to LSCs may aid in target discovery by uncovering pathways critical to initiation, self-renewal, and survival of the CML LSCs. Gene set enrichment analysis (GSEA) [23-25] of all Gene Ontology (GO)[27] and KEGG[28, 29] gene sets was used to identify pathways that show significant and coordinate up or down regulation of pathway components using all genes interrogated by the microarray platform. Significant terms with a q-value (multiple hypothesis testing corrected p-value) less than 0.01 indicated upregulated and downregulated gene sets that are putatively important to LSC biology; these terms were categorized by cellular functions (Supplementary Table 2 shows all significant gene sets). The top three GO and KEGG terms for each category are shown in Figure 3. Gene sets that were upregulated in CML versus normal CD34^+^CD38^−^ALDH^high^ were involved in cell cycle and proliferation, mRNA processing, translation, DNA repair, oxidative metabolism, protein processing, immune response, and metabolic processes. Key downregulated gene sets in the CML CD34^+^CD38^−^ALDH^high^ cells were associated with the cell surface and extracellular matrix, differentiation and developmental programs, cellular response to stimuli, and TGF-β and BMP signaling pathways.

**Figure 4:**
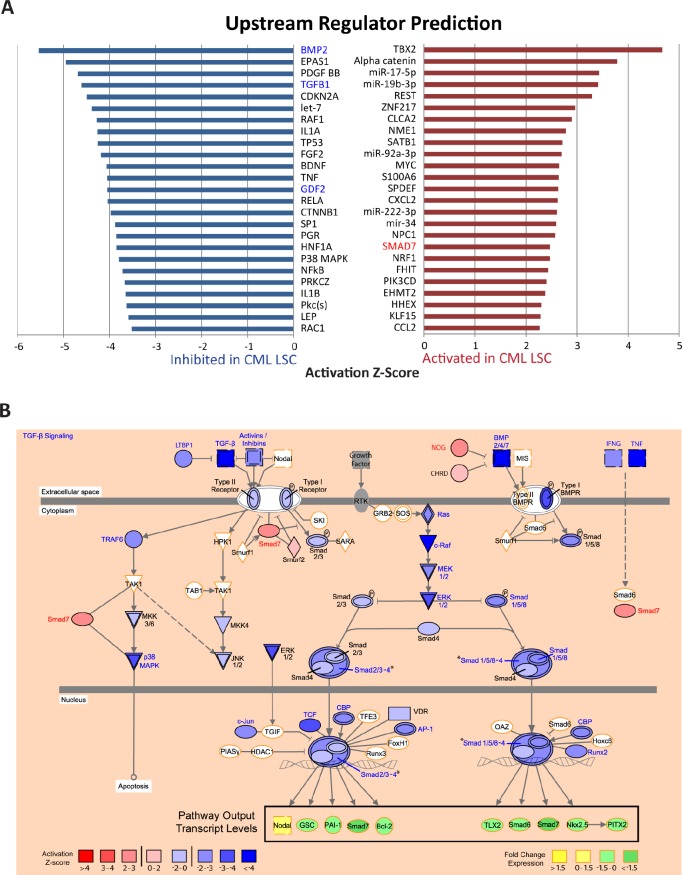
TGF-beta signaling pathway activity is altered in CML LSCs A) The IPA Upstream Regulator analysis was used to identify key regulatory molecules predicted to explain the gene expression differences observed between CML and normal CD34^+^CD38^−^ALDH^high^ cells. Activation Z-scores were calculated for each candidate regulator. Upstream regulators with Z-scores > 2 were considered to be activated (red bars) in CML CD34^+^CD38^−^ALDH^high^ cells. Those with Z-scores < -2 were considered to be inhibited (blue bars) in CML CD34^+^CD38^−^ALDH^high^ cells. Names of activated or inhibited TGF-β pathway members are distinguished in red or blue text, respectively. B) TGF-β signaling pathway. Activated upstream regulators are colored in red; inhibited, blue. Red to blue gradient denotes Z-score value. Activated molecules with a significant Z-score > 2 are distinguished with red text; Inhibited molecules with a significant Z-score < -2, blue text; upstream regulators with a Z-score between -2 and 2, black text. White molecules with orange outline are not considered upstream regulators by IPA. Shape of molecule corresponds to molecule type, as described (Ingenuity® Systems, www.ingenuity.com). A group of molecules with similar functions, depicted by a slash (/) in group name, is colored by a representative molecule with the greatest absolute value Z-score. *indicates a complex of upstream regulators where the activity of the complex is dependent on the activity of all molecules represented. In this case, a separate Z-score was assigned for the complex as a whole and is colored accordingly. Fold change expression values of pathway output transcripts are colored by yellow to green gradient. Yellow indicates upregulation and green, downregulation, of gene expression observed by differential expression analysis of microarray, as discussed in “Methods”.

One challenge in interpreting the results of the gene-set enrichment analyses is that, for many molecular pathways, there may be a de-coupling between the transcriptional levels of the pathway components and the steady-state downstream output of the pathways, often due to complex feedback mechanisms. Therefore, it would be useful to directly examine whether the steady state transcriptional output of the pathway is consistent with overall pathway activation or inactivation. To carry out this type of vectoral analysis, we used the IPA Upstream Regulator analysis, which integrates literature-based information on the relationship between a given candidate upstream regulator and the direction of its influence on the transcriptional level of each of its downstream targets with the differential expression data generated in a given experiment to predict the activation (or inactivation) state of the upstream regulator (Ingenuity® Systems, www.ingenuity.com). Each candidate upstream regulator was assigned a Z-score, representing the confidence with which the regulator is activated or inactivated, with high positive Z-scores representing activation and high negative Z-scores representing inactivation of the function of each upstream regulator. We applied this analysis to our gene expression data from CML and normal CD34^+^CD38^−^ALDH^high^ cells. A Z-score greater than 2 or less than -2 was considered to be activated or inhibited, respectively, in CML relative to normal CD34^+^CD38^−^ALDH^high^ cells (Supplementary Table 3 shows all significant molecules, excluding all “chemical”-related upstream molecule types). The top upstream regulator molecules showed activation of several oncogenes, such as *MYC*, *TBX2*, and *CCND1*, and inflammatory chemokines, such as *CCL2* and *CXCL2*, and inhibition of several tumor suppressors, including *TP53* and *CDKN2A* (Figure 4A, excluding “chemical”-related and “other” upstream molecule types; Supplementary Table 3). Consistent with downregulation of the TGF-β/BMP pathways as observed by GSEA, we observed a strong inhibition of the transcriptional output of the TGF-β and BMP signaling pathway (Figure 4B), with inhibition of pathway agonists including *TGFB1* itself, *BMP2*, *GDF2*, and activation of pathway antagonists, such as *SMAD7* and *NOG* (Figure 4A,B).

### Alternative transcriptional isoforms in CML LSCs

**Figure 5:**
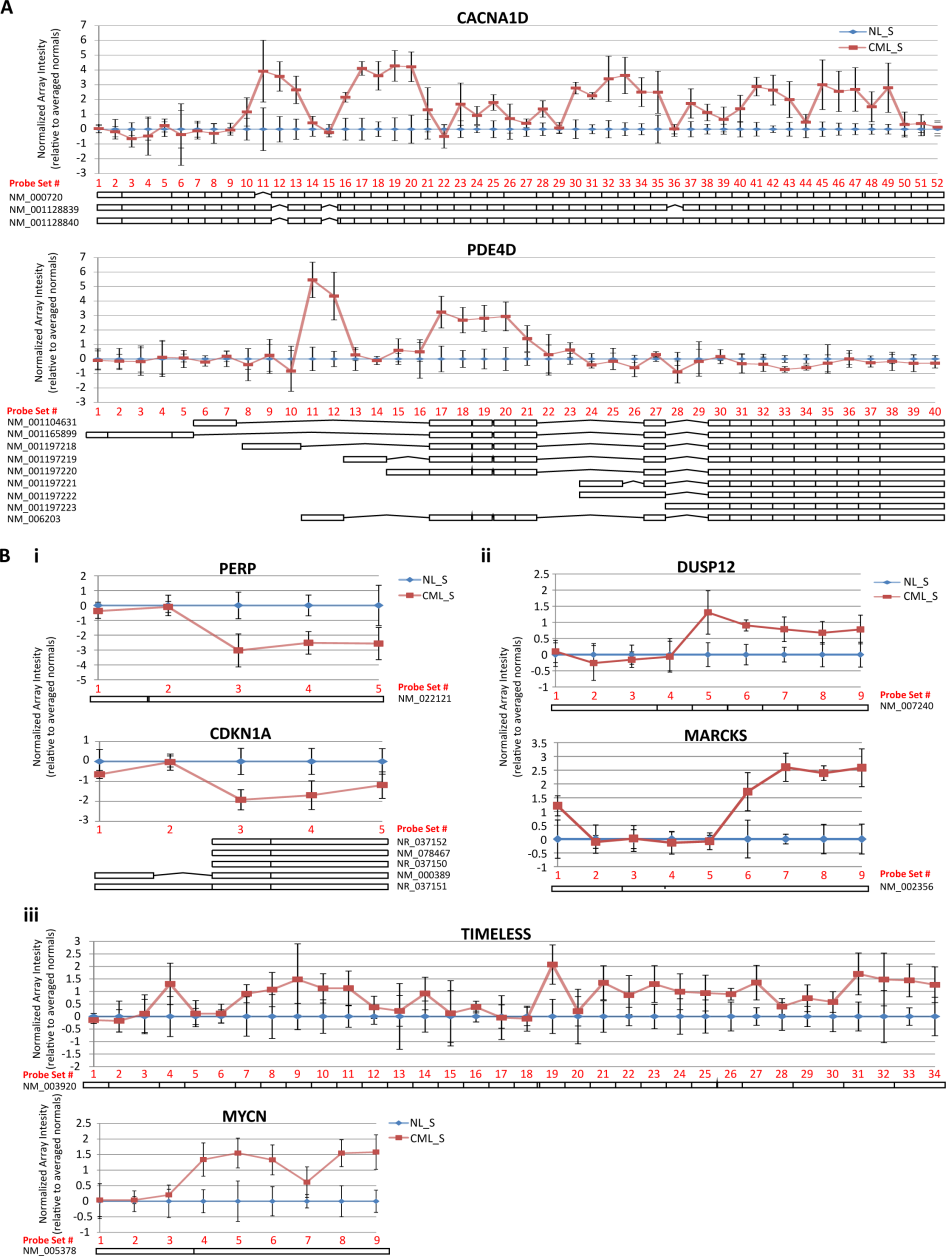
Exon-level analysis reveals evidence of alternative splicing in CML LSCs A) Exon-level microarray data was analyzed for evidence of alternative transcript expression by ANOVA using the default conditions on the Partek alternative transcript workflow. Genes with an alternative splicing p-value < 0.01 were considered significant. The top two genes with the most significant alternative splicing p-values are shown. For each probeset within a gene, the log_2_(normalized intensities) for each sample was adjusted by the average normalized intensity of the normal samples. The resulting mean and standard deviation for CML or normal samples was plotted according to probeset number, assigned 5'-3', for each representative gene. B) Functional annotation of genes with a p(alternative splicing) < 0.01 revealed that alternative transcripts in CML compared to normal CD34^+^CD38^−^ALDH^high^ cells were enriched for genes involved in pathways commonly altered in cancer; (i) p53 signaling, (ii) kinase binding, (iii) cell proliferation. Plots were constructed based on the human reference genome hg19 (GRCh37). Schematics of known refseq transcriptional isoforms are positioned below each graph and are drawn with respect to location of probesets interrogating each exon/intron.

Gene sets associated with RNA processing and, more specifically, mRNA processing were shown by GSEA to be significantly differentially regulated in the CML compared to normal CD34^+^CD38^−^ALDH^high^ cells (Figure 3). We, therefore, examined the exon array data to explore differential exon usage in the CML versus normal CD34^+^CD38^−^ALDH^high^ cells. Evidence of alternative splicing, defined for a given gene as one or more exons displaying expression patterns different from the behavior of the other exons, was apparent in 236 genes (FDR = 0.01; Supplementary Table 4). The top two genes ranked by alternative spicing p-value that showed unique exon behavior were *CACNA1D* and *PDE4D* (Figure 5A). *CACNA1D* also was identified as a top upregulated gene in CML stem and/or progenitor populations compared to normal (Figure 2). This differential expression was probably due to extensive alterations in exon usage across the gene, whereas *PDE4D* displayed preferential expression of specific alternative transcript isoforms in CML CD34^+^CD38^−^ALDH^high^ cells compared to their normal counterparts. Functional annotation of this alternatively transcribed gene list by DAVID analysis was done to gain further insight into the biological processes affected by alternative exon usage/alternative splicing in CML CD34^+^CD38^−^ALDH^high^ cells. This analysis revealed that alternative transcripts in the CML CD34^+^CD38^−^ALDH^high^ cells, when compared to normal counterparts, were enriched in cellular proliferation genes, p53 signaling pathway, and kinase binding genes. There were 29 genes identified to be involved in regulation of cellular proliferation, including *MYCN* and *TIMELESS* (Figure 5Biii). Seven genes were involved in p53 signaling, including *CDKN1A*, which was also found in the cell proliferation category, and *PERP* (Figure 5Bi). Twelve genes had kinase binding functions, including *MARCKS* and *DUSP12* (Figure 5Bii).

## DISCUSSION

LSCs appear to persist in most CML patients on TKIs, and the persistence of these cells remains a major obstacle to cure.[5, 7, 9] We previously reported that ALDH expression enriched for CD34^+^CD38^−^ cells capable of engrafting NSG mice from normal marrow[18] as well as CML[20], thus, presumably representing the primitive stem cell fractions in both. Moreover, expression of some putative targets by the CML LSCs differed significantly from that of the more prevalent progenitor cells[20], highlighting the need to study refined LSC populations. Additionally, other CML antigens were expressed at comparable levels to normal stem/progenitor cells, suggesting a lack of leukemia-specificity and a high likelihood that therapies targeting these candidates might cause undue toxicity to normal hematopoiesis[20].

We employed exon microarray technology to perform whole transcriptome analysis of highly enriched CP CML and normal stem and progenitor cell populations with the goal of identifying unique putative LSC targets. Interestingly, principal components analysis revealed that expression patterns were remarkably similar between the CD34^+^CD38^−^ALDH^high^ cells from CML patients and those from normal donors. In fact, the similarities were greater than those observed between the CML LSCs (CD34^+^CD38^−^ALDH^high^ cells) and the CML progenitors (CD34^+^CD38^+^ cells), underpinning the challenge in selectively targeting LSCs without injuring normal HSCs. Nonetheless, the comprehensive approach and highly refined populations utilized in this analysis allowed identification of important new putative LSC targets that were more highly expressed by the CML LSC and/or progenitor cell fractions compared to normal stem/progenitor cell fractions.

A significant number of genes over-expressed in CML LSCs compared to their normal counterparts encoded cell surface proteins, including, *IL2Rα*, *DPP4*, *PTPRD*, *CACNA1D*, *IL1RAP*, *SLC4A4*, and *KCNK5*. The surface location of these candidates may render them particularly vulnerable to targeting by immune-based strategies. *DPP4*, also known as CD26, encodes dipeptidyl peptidase 4, and is especially interesting as a possible target for LSC-directed therapy. One of the known targets of its peptidase cleavage activity is CXCL12[30], and upregulation of DPP4 on the surface of CML LSCs may allow these cells to escape the homing/niche interactions imposed by the CXCL12/CXCR4 chemokine-receptor system[31], leading to dysregulated LSC growth and survival. Therefore, drugs capable of inhibiting the DPP4 dipeptidyl peptidase catalytic activity, which are currently FDA-approved for treatment of diabetes[32], may have utility in targeting CML LSCs. IL2RA is also a particularly attractive LSC target since multiple biologic agents directed against it are currently under clinical investigation[33]. IL1RAP has been identified previously as a putative therapeutic stem cell-specific target in CML[34], as well as in acute myeloid leukemia (AML) and myelodysplastic syndrome (MDS) patients, with high expression correlating with poor overall survival in AML[35]. Similarly, in this study, we identified *IL1RAP* upregulation on CML LSCs; the availability of IL-1 receptor antagonists or decoy receptors that are currently FDA-approved for the treatment of several inflammatory disorders[36] may allow effective targeting of the CML LSC. Among the other genes found to be upregulated in LSCs compared to normal HSCs, *BLM*,,*KYNU*, *PTPRD*, *RAB31*, and *HPGDS* are known to have enzymatic activities involved in key signaling and metabolic pathways; development of inhibitors of these enzymes may allow LSC targeting.

Taking advantage of the comprehensive coverage of the exon array platform, we also identified several genes that were dysregulated in LSCs at the level of alternative transcriptional isoforms and alternative exon usage. Interestingly genes showing alternative splicing were enriched in p53 signaling, protein kinase binding and cell proliferation. Therefore, alternative splicing may account in part for the increased cell proliferation, resistance to apoptosis, and dysregulated kinase signaling characteristic of CML[37]. It is expected that these pathways and their components are susceptible to pharmacologic inhibition. Of particular interest, the cyclic-AMP specific phosphodiesterase, *PDE4D*, was found to be upregulated in CML LSCs compared to normal HSCs by preferential expression of a specific alternative transcript isoform. Likewise, the dual specificity phosphatase, *DUSP12*, and the voltage-dependent L-type calcium channel, *CACNA1D*, appear to become upregulated in CML LSCs via alternative exon usage. It is possible that alternative splicing of *DUSP12* in CML LSCs could underlie immunogenic responses that seem to correlate with improved survival after donor lymphocyte infusion[38]. Although PDE4 inhibitors and L-type calcium channel blockers are available, development of isoform specific inhibitors may aid in CML LSC targeting. Therefore, such alternative transcription analyses could be used to identify functionally critical exons and their corresponding protein domains for development of targeted and immunomodulatory therapies.

Using these comprehensive transcriptome data, we were able to identify key pathways that were altered in the LSCs compared to normal HSCs. Consistent with previous findings of Bruns et al [39], we observed upregulation of several pathways involved in cell proliferation/cell cycle, and downregulation of pathways involved in cell surface interactions, development, and differentiation. These pathway alterations may underlie the increased cell proliferation and resistance to apoptosis that are characteristic of CML and may also play a significant role in recognized resistance mechanisms of LSCs, such as dysregulation of niche interactions, cell cycle, survival, self-renewal, and metabolism. Interestingly, and somewhat unexpectedly, we also observed upregulation of pathways involved in oxidative metabolism, suggesting that LSCs may not be as metabolically quiescent as previously thought[40]. The accompanying upregulation of DNA repair pathways in the CML LSCs may indicate a requirement for guarding against DNA damage induced by a potential increase in production of reactive oxygen species during oxidative metabolism. Additionally, we identified a number of signaling pathways that showed evidence of activation in the LSCs. Particularly interesting are the targets with specific inhibitors already under clinical investigation, including a neutralizing monoclonal antibody to CCL2[41] and cyclin dependent kinase 4/6 inhibitors, inhibiting activation by partnering cyclin CCND1[42, 43]. Additionally, we found that the TGF-β/BMP pathway was coordinately downregulated in the CML LSC compared to normal HSC, and pathway antagonists, such as *SMAD7* were highly activated. The likely contribution of *SMAD7* activation to the observed TGF-β pathway inhibiton in CML LSCs compared to normal HSCs and the current clinical investigation of antisense oligonucleotides for *SMAD7* inhibition in Crohn's disease[44] make it an attractive target for CML LSC-directed therapy. Although previous reports have shown that the TGF-β pathway is critical for CML LSC survival[45-47], it also has been suggested that TGF-β has a dual role in tumor progression, acting as a tumor suppressor in the very early stages of tumorigenesis[48, 49].

We have developed an important resource for identifying the gene expression changes, pathway alterations, and alternative exon usage that can allow selective targeting of CP CML LSCs. Some of these targets, such as *IL2RA* and *DPP4*, may be amenable to immediate clinical translation with currently available therapies. While this work requires further functional validation and target credentialing, it offers the promise of LSC-targeted therapies, which may prove curative in CML while minimizing harm to normal hematopoiesis.

## METHODS

### Patient and normal donor bone marrow specimens, enrichment of stem and progenitor cell populations, and nucleic acid extraction

Bone marrow was obtained from 5 patients with newly-diagnosed and untreated CP CML, as well as from 5 healthy bone marrow donors. Informed consent was obtained from all patients and healthy donors prior to sample collection in accordance with the Declaration of Helsinki, under a research protocol approved by the Johns Hopkins Institutional Review Board. CD34^+^CD38^−^ALDH^high^ stem cells and CD34^+^CD38^+^ progenitor cells were collected from each marrow specimen as described previously[20]. Briefly, CD34^+^ cells were selected using Miltenyi Biotec (Auburn, CA) microbeads (binding the class II CD34 epitope) followed by column enrichment per the manufacturer's recommendations. These cells were then stained with Aldefluor (Aldagen, Durham, NC) to assess ALDH activity, phycoerythrin-conjugated anti-CD34 antibodies (binding the class III CD34 epitope), and allophycocyanin-conjugated anti-CD38 antibodies (BD Biosciences, San Jose, CA), and sorted using a MoFlo cell sorter (Beckman Coulter) into CD34^+^CD38^−^ALDH^high^ and CD34^+^CD38^+^ fractions. DNA and RNA were extracted from at least 50,000 cells from each population using the All-prep micro kit (Qiagen, Valencia, CA, USA).

### Fluorescence in situ hybridization (FISH)

Isolation of leukemic cells was confirmed by FISH for *BCR-ABL* on cytospins of each sorted cell fraction, fixed in 3:1 Methanol: Glacial Acetic acid (Sigma-Aldrich, St. Louis, MO, USA). FISH was performed by the Johns Hopkins Cytogenetics Core, using the Vysis LSI *BCR-ABL* Dual Color, Dual Fusion translocation probe (Abbot Molecular, Des Plaines, IL, USA) per manufacturer's instructions. Slides were analyzed on a fluorescence microscope with a triple-band pass filter for DAPI, Spectrum Orange, and Spectrum Green.

### Gene expression microarrays and analysis

Total RNA from sorted cell populations was subjected to cDNA synthesis and linear amplification using the Ovation RNA Exon Module amplification system (NuGEN, San Carlos, CA) according to the manufacturer's protocols. The resulting material was then fragmented and biotin-end-labeled using the Encore Biotin Module (NuGEN) and hybridized to Human Exon 1.0 ST whole genome gene expression microarrays (Affymetrix, Santa Clara, CA) according to the manufacturer's protocols at the Johns Hopkins Microarray facility. The microarray gene expression data was analyzed with Partek Genomic Suite software (http://www.partek.com/partekgs) using the exon array workflow with default conditions (data imported and normalized using log_2_ transformation, default RMA background correction and normalization of core meta-probe sets) unless otherwise specified. Gene expression summaries from the imported normalized intensity data was subjected to principal components analysis. Two-way analysis of variance (ANOVA) of gene summary data was performed to find differentially expressed genes between all cell populations, focusing on the contrasts between CML versus normal samples and CML CD34^+^CD38^−^ALDH^high^ versus normal CD34^+^CD38^−^ALDH^high^ populations. Genes with |log_2_(fold-change)| > 1 and false discovery rate (FDR) of 0.05 were identified as significantly differentially expressed. A gene list specifically focusing on contrasts between CML and normal CD34^+^CD38^−^ALDH^high^ cells with |log_2_(fold-change)| > 1 and false discovery rate (FDR) of 0.05 was uploaded to the Database for Annotation, Visualization, and Integrated Discovery (DAVID) v6.7 (http://david.abcc.ncifcrf.gov/) for functional annotation analyses[21, 22] of enriched gene ontology (GO) and KEGG pathway terms. Lists comprised of all arrayed genes with expression data from the CML versus normal CD34^+^CD38^−^ALDH^high^ comparison were subjected to gene-set enrichment analysis (GSEA), as described previously[23-25], or directly uploaded into Ingenuity Pathway Analysis (IPA) software (Ingenuity® Systems, June 2012, www.ingenuity.com). For GSEA, all GO and KEGG terms with a q-value less than 0.01 were considered significant. Core analysis was run in IPA utilizing all default settings, with exception of the Human Exon 1.0 ST array as the reference gene set. This analysis generated a list of potential upstream transcriptional regulators and predicted the activity of each by calculation of overlap p-value using a Fisher's Exact test and the activation Z-score as described (Ingenuity® Systems, www.ingenuity.com). Calculations were based on known interactions between the predicted upstream transcriptional regulators and their downstream target gene set according to the Ingenuity® Knowledge Base and measured expression changes in the array data set. Upstream regulators with |z-score| > 2.00 were nominated as significant, with a positive Z-score representing activation and a negative value, inhibition. The list of upstream regulators and activation z-score values were also utilized to assign the activation state of each component of the TGF-β pathway, which was defined using the IPA and KEGG pathway map data (http://www.genome.jp/kegg/pathway.html). The raw and normalized data are available from the Gene Expression Omnibus (GEO) with accession number GSE43754. For alternative transcript analysis, exon level microarray data from the CML and normal CD34^+^CD38^−^ALDH^high^ RNA was subjected to ANOVA analysis using the default conditions on the Partek alternative transcript workflow. Genes with alternative transcript p-value < 0.01 were subjected to analysis with DAVID v.6.7 as described above. For each probeset within a gene, the log_2_(normalized intensities) for each sample was adjusted by the average normalized intensity of the normal samples. The resulting mean and standard deviation for CML or normal samples was plotted according to probeset number, assigned 5'-3', for each representative gene.

### Real-time reverse transcriptase polymerase chain reaction

Excess extracted RNA from patient samples was used to synthesize cDNA using SuperScript® III Reverse Transcriptase (RT) (Invitrogen, Carlsbad, CA). Newly synthesized cDNA from unamplified RNA or excess amplified cDNA prior to labeling and array hybridization from each CML and normal patient sample was used to validate array results by quantitative RT-PCR of *GAS2*, *DPP4*, *CDH2*, *IL2RA*, *GAPDH* and *ACTB* using the iQ Supermix (Bio-Rad, Hercules, CA) and gene-specific TaqMan® assays (Life Technologies Co., Carlsbad, CA). The relative amount of the gene of interest was determined using the ΔΔ*C*_t_ method, relative to the average expression of all samples for that gene and *GAPDH* expression for *GAS2*, *DPP4*, and *CDH2* or *ACTB* for *IL2RA*. Quantitative RT-PCR results from amplified starting material or SuperScript® III converted unamplified cDNA were compared for the gene *IL2RA* and showed consistent results. The remaining genes were verified using amplified starting material only. All quantitative PCR experiments were done in duplicate.

## CML Gerber et al_Supp Tables


