# Supplementary material for: Genome-wide comparison of the transcriptomes of highly enriched normal and chronic myeloid leukemia stem and progenitor cell populations
Source: Oncotarget. 2013 May 6;4(5):715–28. doi: 10.18632/oncotarget.990 (PMC3742832; doi:10.18632/oncotarget.990)
Supplement: Supplementary file 1 [file oncotarget-04-715-s001.pdf]

Supplementary Table 1. Chronic Phase CML and Normal Donor Bone Marrow Samples

			% *BCR-ABL* Positive Cells	
Sample ID	Diagnosis	Age	Stem ($CD34^+CD38^-ALDH^{high}$)	Progenitor ($CD34^+CD38^+$)
NL1	Normal	42	NA	NA
NL2	Normal	56	NA	NA
NL3	Normal	60	NA	NA
NL4	Normal	57	NA	NA
NL5	Normal	60	NA	NA
CML1	Chronic Phase CML	41	100.0%	98.5%
CML2	Chronic Phase CML	28	98.5%	98.0%
CML3	Chronic Phase CML	55	99.5%	98.5%
CML4	Chronic Phase CML	50	32.0%	96.0%
CML5	Chronic Phase CML	69	80.5%	99.0%

Stem and progenitor cells from bone marrow samples of 5 normal donors and 5 CP CML patients were isolated, and the presence of *BCR-ABL* $^+$ leukemic cells in CP CML samples was confirmed by FISH on cytospin preparations, as described in "Methods".

NA indicates not applicable.

Supplementary Table 2A. Upregulated Gene Sets in CML Compared to Normal Stem Cells

Category*	GO ID	GO/KEGG Term	*P*	*Q* †	Transcript Clusters‡
Cell Cycle/Proliferation	GO:0007049	cell cycle	<1E-15	<1E-15	511
	GO:0051301	cell division	<1E-15	<1E-15	276
	GO:0007067	mitosis	<1E-15	<1E-15	220
	GO:0006260	DNA replication	<1E-15	2.00E-15	170
	KEGG	DNA replication reactome	1.24E-12	7.42E-11	42
	GO:0007059	chromosome segregation	3.47E-13	1.40E-10	57
	GO:0000775	chromosome, centromeric region	7.25E-12	2.39E-09	62
	KEGG	cell cycle KEGG	1.99E-08	2.98E-07	89
	GO:0000777	condensed chromosome kinetochore	9.66E-09	1.84E-06	67
	GO:0004386	helicase activity	2.66E-08	4.19E-06	115
	GO:0051436	negative regulation of ubiquitin-protein ligase activity involved in mitotic cell cycle	3.19E-08	4.83E-06	65
	GO:0031145	anaphase-promoting complex-dependent proteasomal ubiquitin-dependent protein catabolic process	3.59E-08	5.22E-06	66
	GO:0000922	spindle pole	5.08E-08	6.83E-06	56
	GO:0017111	nucleoside-triphosphatase activity	1.81E-07	2.12E-05	63
	GO:0051437	positive regulation of ubiquitin-protein ligase activity involved in mitotic cell cycle	3.66E-07	3.80E-05	67
	KEGG	G1 to S cell cycle reactome	5.53E-06	5.53E-05	72
	GO:0006270	DNA-dependent DNA replication initiation	6.04E-07	6.08E-05	16
	GO:0005813	centrosome	5.48E-06	4.42E-04	138
	GO:0004003	ATP-dependent DNA helicase activity	6.26E-06	4.83E-04	25
	GO:0000793	condensed chromosome	6.64E-06	5.02E-04	19
	GO:0005814	centriole	1.44E-05	1.00E-03	42
	GO:0005876	spindle microtubule	1.64E-05	1.12E-03	31
	GO:0008026	ATP-dependent helicase activity	2.69E-05	1.74E-03	64
	GO:0000723	telomere maintenance	3.13E-05	1.96E-03	23
	GO:0005663	DNA replication factor C complex	4.66E-05	2.60E-03	6
	GO:0008094	DNA-dependent ATPase activity	5.28E-05	2.86E-03	34
	GO:0005819	spindle	5.94E-05	3.13E-03	101
	GO:0000796	condensin complex	7.44E-05	3.81E-03	6
	GO:0007080	mitotic metaphase plate congression	9.13E-05	4.54E-03	12
	GO:0003678	DNA helicase activity	9.71E-05	4.68E-03	20
	GO:0016568	chromatin modification	1.20E-04	5.58E-03	217
	GO:0000070	mitotic sister chromatid segregation	1.51E-04	6.62E-03	12
	GO:0003689	DNA clamp loader activity	1.76E-04	7.51E-03	5
	GO:0007093	mitotic cell cycle checkpoint	2.12E-04	8.64E-03	14
	GO:0000940	condensed chromosome outer kinetochore	2.38E-04	9.53E-03	10
	GO:0006275	regulation of DNA replication	2.39E-04	9.53E-03	9
	GO:0000075	cell cycle checkpoint	2.50E-04	9.85E-03	17
mRNA Processing	GO:0008380	RNA splicing	2.90E-14	1.33E-11	281
	KEGG	mRNA processing reactome	9.68E-10	2.90E-08	103
	GO:0005681	spliceosomal complex	7.47E-09	1.51E-06	127
	GO:0006397	mRNA processing	1.07E-07	1.34E-05	247
	KEGG	RNA transcription reactome	2.46E-06	2.96E-05	38
	GO:0006406	mRNA export from nucleus	3.52E-06	3.12E-04	36
	GO:0000184	nuclear-transcribed mRNA catabolic process, nonsense-mediated decay	6.60E-05	3.42E-03	36
Translation	GO:0005730	nucleolus	1.09E-11	3.30E-09	776
	GO:0006364	rRNA processing	1.17E-08	2.02E-06	94
	GO:0006412	translation	4.66E-08	6.50E-06	212
	GO:0005840	ribosome	8.66E-08	1.12E-05	178
	GO:0008033	tRNA processing	1.05E-06	1.03E-04	64
	GO:0005761	mitochondrial ribosome	2.52E-06	2.34E-04	19

Supplementary Table 2A (con't). Upregulated Gene Sets in CML Compared to Normal Stem Cells

Category*	GO ID	GO/KEGG Term	*P*	*Q*†	Transcript Clusters‡
	GO:0003735	structural constituent of ribosome	8.09E-05	4.08E-03	153
DNA Repair	GO:0006281	DNA repair	2.19E-11	5.30E-09	216
	GO:0006297	nucleotide-excision repair, DNA gap filling	1.03E-05	7.31E-04	17
	GO:0006302	double-strand break repair	3.27E-05	1.98E-03	40
	GO:0000731	DNA synthesis involved in DNA repair	3.50E-05	2.08E-03	10
	GO:0006310	DNA recombination	5.86E-05	3.13E-03	64
	GO:0003684	damaged DNA binding	1.93E-04	8.13E-03	47
Oxidative Metabolism	GO:0005743	mitochondrial inner membrane	6.09E-10	1.30E-07	269
	KEGG	electron transport chain	1.60E-08	2.98E-07	81
	GO:0005759	mitochondrial matrix	3.12E-06	2.83E-04	160
	GO:0006120	mitochondrial electron transport, NADH to ubiquinone	3.93E-06	3.40E-04	44
	GO:0022900	electron transport chain	4.54E-06	3.83E-04	100
	GO:0070469	respiratory chain	8.54E-06	6.33E-04	51
	GO:0005747	mitochondrial respiratory chain complex I	1.85E-05	1.24E-03	49
	GO:0008137	NADH dehydrogenase (ubiquinone) activity	3.27E-05	1.98E-03	47
	GO:0006099	tricarboxylic acid cycle	5.02E-05	2.76E-03	23
	KEGG	Krebs-TCA cycle	7.44E-04	4.96E-03	30
	GO:0015992	proton transport	1.45E-04	6.41E-03	40
Protein Processing	GO:0006457	protein folding	1.06E-08	1.93E-06	160
	GO:0051603	proteolysis involved in cellular protein catabolic process	3.37E-07	3.60E-05	28
	GO:0004298	threonine-type endopeptidase activity	5.43E-06	4.42E-04	20
	GO:0003755	peptidyl-prolyl cis-trans isomerase activity	6.00E-06	4.73E-04	38
	GO:0015031	protein transport	1.02E-05	7.31E-04	413
	GO:0000502	proteasome complex	9.80E-05	4.68E-03	56
	GO:0006461	protein complex assembly	1.28E-04	5.90E-03	110
	GO:0030117	membrane coat	1.41E-04	6.30E-03	26
	KEGG	proteasome degradation	1.26E-03	7.54E-03	56
Immune Response	GO:0016446	somatic hypermutation of immunoglobulin genes	4.61E-05	2.60E-03	8
Metabolic Processes	KEGG	cholesterol biosynthesis	2.23E-04	1.67E-03	15
	KEGG	heme biosynthesis	2.03E-04	1.67E-03	8
	GO:0051287	NAD binding	9.79E-05	4.68E-03	37
	GO:0044237	cellular metabolic process	1.36E-04	6.18E-03	19
	GO:0006783	heme biosynthetic process	2.01E-04	8.29E-03	17
Multiple	GO:0005634	nucleus	<1E-15	<1E-15	4938
	GO:0005739	mitochondrion	<1E-15	<1E-15	1295
	GO:0005654	nucleoplasm	<1E-15	<1E-15	511
	GO:0005515	protein binding	1.97E-12	7.16E-10	6651
	GO:0000166	nucleotide binding	1.21E-11	3.38E-09	2012
	GO:0003723	RNA binding	1.30E-11	3.38E-09	598
	GO:0005524	ATP binding	3.50E-11	7.93E-09	1525
	GO:0016787	hydrolase activity	1.66E-08	2.74E-06	962
	GO:0003677	DNA binding	1.66E-07	2.01E-05	1692
	GO:0005737	cytoplasm	2.35E-07	2.67E-05	4680
	GO:0016853	isomerase activity	3.35E-07	3.60E-05	115
	GO:0016874	ligase activity	2.44E-06	2.33E-04	348
	GO:0008201	heparin binding	2.90E-06	8.09E-04	112
	GO:0016740	transferase activity	2.04E-05	1.35E-03	1191
	GO:0016779	nucleotidyltransferase activity	3.12E-05	1.96E-03	73
	GO:0004527	exonuclease activity	4.05E-05	2.37E-03	37
	GO:0016363	nuclear matrix	4.38E-05	2.52E-03	66

Supplementary Table 2A (con't). Upregulated Gene Sets in CML Compared to Normal Stem Cells

Category*	GO ID	GO/KEGG Term	*P*	Q†	Transcript Clusters‡
	GO:0008415	acyl transferase	1.07E-04	5.03E-03	137
	GO:0004519	endonuclease activity	1.58E-04	6.84E-03	60
	GO:0008168	methyltransferase activity	1.96E-04	8.18E-03	120

Supplementary Table 2B. Downregulated Gene Sets in CML Compared to Normal Stem Cells

Category*	GO ID	GO/KEGG Term	*P*	Q†	Transcript Clusters‡
Cell Surface/ECM	GO:0005886	plasma membrane	<1E-15	<1E-15	2879
	GO:0005576	extracellular region	<1E-15	<1E-15	1739
	GO:0005615	extracellular space	6.10E-14	7.38E-11	746
	GO:0016021	integral to membrane	1.27E-12	1.15E-09	3853
	GO:0007155	cell adhesion	3.30E-08	1.71E-05	501
	GO:0005887	integral to plasma membrane	1.52E-06	4.59E-04	1020
	GO:0045202	synapse	3.65E-06	8.86E-04	252
	GO:0005578	proteinaceous extracellular matrix	3.66E-06	8.86E-04	220
	GO:0007156	homophilic cell adhesion	3.97E-06	8.99E-04	106
	GO:0030054	cell junction	4.83E-06	1.03E-03	401
	GO:0031225	anchored to membrane	4.02E-05	5.83E-03	118
Differentiation/Development Programs	GO:0007275	multicellular organismal development	1.00E-09	7.28E-07	886
	GO:0030154	cell differentiation	1.15E-06	3.79E-04	472
	KEGG	nuclear receptors	1.83E-05	5.50E-04	40
	GO:0001501	skeletal system development	1.77E-05	3.06E-03	132
	GO:0007507	heart development	3.12E-05	4.71E-03	127
Cellular Response to Stimuli	GO:0004872	receptor activity	2.61E-09	1.58E-06	1184
	GO:0005509	calcium ion binding	1.04E-07	4.73E-05	606
	GO:0043565	sequence-specific DNA binding	2.63E-07	1.06E-04	459
	GO:0050896	response to stimulus	5.86E-07	2.13E-04	485
	GO:0045944	positive regulation of transcription from RNA polymerase II promoter	5.18E-06	1.04E-03	320
	GO:0003700	sequence-specific DNA binding transcription factor activity	8.06E-06	1.54E-03	936
	GO:0008083	growth factor activity	2.45E-05	4.04E-03	161
	GO:0003705	sequence-specific distal enhancer binding RNA polymerase II transcription factor activity	2.94E-05	4.65E-03	78
Signaling Pathways	KEGG	TGF-beta signaling pathway	1.19E-05	5.50E-04	47
	GO:0030509	BMP signaling pathway	1.54E-05	2.79E-03	46

GO and KEGG terms with a q-value < 0.01 are included in the table.

*Common cellular process among a group of associated GO and KEGG terms.

†Q-value represents the false discovery rate of the p-value, as previously described[50].

‡Indicates the number of transcripts within the data set that fall within the specific GO or KEGG term.

Supplementary Table 3. Predicted Upstream Regulator Molecules in CML Compared to Normal Stem Cells

Upstream Regulator*	Fold Change (Expression)†	Molecule Type	Predicted Activation State‡	Activation Z-score§	*P* of overlap||
BMP2	-1.256	growth factor	Inhibited	-5.532	4.21E-04
EPAS1	-1.141	transcription regulator	Inhibited	-4.946	2.07E-04
PDGF BB	NA	complex	Inhibited	-4.686	1.09E-05
TGFB1 (includes EG:21803)	-1.080	growth factor	Inhibited	-4.610	9.84E-19
CDKN2A	1.034	transcription regulator	Inhibited	-4.492	2.00E-04
BNIP3L	1.050	other	Inhibited	-4.451	4.55E-02
let-7	NA	microRNA	Inhibited	-4.382	1.22E-02
RAF1	1.051	kinase	Inhibited	-4.279	8.94E-05
IL1A	-1.340	cytokine	Inhibited	-4.266	4.40E-03
TP53 (includes EG:22059)	1.245	transcription regulator	Inhibited	-4.256	4.19E-10
FGF2	1.411	growth factor	Inhibited	-4.186	8.59E-06
BDNF	-1.163	growth factor	Inhibited	-4.062	2.49E-03
TNF	1.205	cytokine	Inhibited	-4.049	9.28E-10
GDF2	-1.226	growth factor	Inhibited	-4.048	7.48E-02
RELA	-1.257	transcription regulator	Inhibited	-4.040	3.92E-03
CTNNB1	-1.275	transcription regulator	Inhibited	-3.975	7.64E-07
SP1	-1.027	transcription regulator	Inhibited	-3.879	3.23E-04
PGR (includes EG:18667)	-1.128	ligand-dependent nuclear receptor	Inhibited	-3.853	1.59E-03
HNF1A	1.034	transcription regulator	Inhibited	-3.850	2.01E-10
P38 MAPK	NA	group	Inhibited	-3.792	3.71E-06
NFkB (complex)	NA	complex	Inhibited	-3.716	9.58E-06
PRKCZ	-1.278	kinase	Inhibited	-3.664	7.50E-02
IL1B	2.622	cytokine	Inhibited	-3.647	1.38E-05
Pkc(s)	NA	group	Inhibited	-3.627	6.04E-03
LEP	1.490	growth factor	Inhibited	-3.589	3.02E-04
RBL2 (includes EG:100331892)	-1.551	other	Inhibited	-3.525	2.48E-01
RAC1	-1.008	enzyme	Inhibited	-3.513	1.10E-02
NEUROG3	1.003	transcription regulator	Inhibited	-3.506	2.44E-02
MAPK1	1.075	kinase	Inhibited	-3.504	5.08E-03
IRGM	NA	other	Inhibited	-3.499	1.61E-01
BMP6	-3.460	growth factor	Inhibited	-3.424	2.02E-02
Rb	NA	group	Inhibited	-3.424	1.33E-01
Secretase gamma	NA	complex	Inhibited	-3.393	6.79E-01
EDN1	-2.110	cytokine	Inhibited	-3.378	1.09E-04
EGR1	1.126	transcription regulator	Inhibited	-3.332	3.26E-03
Tgf beta	NA	group	Inhibited	-3.323	9.25E-04
BMP4	-1.609	growth factor	Inhibited	-3.319	5.03E-03
I kappa b kinase	NA	complex	Inhibited	-3.300	3.62E-01
GDNF	-1.451	growth factor	Inhibited	-3.290	1.25E-01
BMPR1A	-1.041	kinase	Inhibited	-3.221	1.09E-01
MAP3K1	NA	kinase	Inhibited	-3.194	8.11E-03
F2	-1.057	peptidase	Inhibited	-3.192	3.85E-04
ETS2	1.235	transcription regulator	Inhibited	-3.174	1.51E-01
HNF4A	1.062	transcription regulator	Inhibited	-3.138	3.09E-23
SIX5	1.052	transcription regulator	Inhibited	-3.134	6.68E-01
OSM	1.059	cytokine	Inhibited	-3.121	8.09E-07
MAPK14	1.033	kinase	Inhibited	-3.070	8.93E-03
IKBKG	1.085	kinase	Inhibited	-3.068	2.25E-01
LDL	NA	complex	Inhibited	-3.066	5.68E-06
GC-GCR dimer	NA	complex	Inhibited	-3.062	2.74E-01
TAF4B	NA	transcription regulator	Inhibited	-3.052	6.18E-01
RB1	1.342	transcription regulator	Inhibited	-3.008	5.31E-05
Calcineurin protein(s)	NA	complex	Inhibited	-3.004	2.15E-01
Ap1	NA	complex	Inhibited	-3.000	8.59E-02
CTNNβ-TCF/LEF	NA	complex	Inhibited	-3.000	4.35E-01
TNFRSF11A	-1.081	transmembrane receptor	Inhibited	-2.985	3.97E-01
Nfat (family)	NA	group	Inhibited	-2.971	1.70E-01
MAP2K1	-1.230	kinase	Inhibited	-2.965	6.30E-04
LEF1	1.069	transcription regulator	Inhibited	-2.947	1.04E-01
TRIM21	1.363	enzyme	Inhibited	-2.947	4.35E-01
SMAD1	-1.024	transcription regulator	Inhibited	-2.944	6.23E-02
TGFB3	-1.090	growth factor	Inhibited	-2.943	9.59E-04
MAP2K5	-1.171	kinase	Inhibited	-2.939	6.46E-01
IL6	1.023	cytokine	Inhibited	-2.932	4.69E-08
FXR ligand-FXR-Retinoic acid-RXRα	NA	complex	Inhibited	-2.930	1.09E-01
EGF (includes EG:13645)	-1.142	growth factor	Inhibited	-2.927	1.22E-05
SMARCB1	1.512	transcription regulator	Inhibited	-2.922	1.13E-02
hCG	NA	complex	Inhibited	-2.922	1.15E-05
CRX	1.026	transcription regulator	Inhibited	-2.918	1.19E-01
AVP	-1.145	other	Inhibited	-2.908	3.92E-02
Creb	NA	group	Inhibited	-2.892	4.40E-03
TCF3	1.046	transcription regulator	Inhibited	-2.877	3.10E-04
Mapk	NA	group	Inhibited	-2.861	5.08E-02
CHUK	1.113	kinase	Inhibited	-2.817	4.96E-03
PTH	-1.004	other	Inhibited	-2.813	9.52E-03
NMDA Receptor	NA	complex	Inhibited	-2.813	1.00E00

Supplementary Table 3 (con't). Predicted Upstream Regulator Molecules in CML Compared to Normal Stem Cells

Upstream Regulator*	Fold Change (Expression)†	Molecule Type	Predicted Activation State‡	Activation Z-score§	*P* of overlap||
USF1	1.178	transcription regulator	Inhibited	-2.799	1.64E-02
WWTR1	-1.315	transcription regulator	Inhibited	-2.789	1.00E00
ARNT	-1.000	transcription regulator	Inhibited	-2.787	1.17E-01
PDGFRB	1.011	kinase	Inhibited	-2.784	4.77E-01
ELK1	1.078	transcription regulator	Inhibited	-2.763	5.18E-02
Smad2/3-Smad4	NA	complex	Inhibited	-2.762	2.50E-01
HIF1A	-1.114	transcription regulator	Inhibited	-2.755	4.34E-03
NTF4	-1.162	growth factor	Inhibited	-2.749	2.74E-01
SHH	1.027	peptidase	Inhibited	-2.747	2.83E-03
PTHLH	1.174	other	Inhibited	-2.721	2.05E-02
NPPC	1.083	other	Inhibited	-2.714	3.62E-01
ABCB6	1.152	transporter	Inhibited	-2.706	1.57E-01
IKBKB	-1.014	kinase	Inhibited	-2.702	1.36E-02
GAL	-1.214	other	Inhibited	-2.698	3.62E-01
Crem	NA	other	Inhibited	-2.697	5.89E-02
TGFA	1.042	growth factor	Inhibited	-2.680	1.46E-01
PTGS2	-1.497	enzyme	Inhibited	-2.675	3.99E-04
SELPLG	1.851	other	Inhibited	-2.646	2.61E-01
GCK	-1.061	kinase	Inhibited	-2.635	9.02E-02
CYBA	1.079	enzyme	Inhibited	-2.630	5.23E-01
ATF2	1.120	transcription regulator	Inhibited	-2.626	1.63E-01
MYOD1	1.022	transcription regulator	Inhibited	-2.624	3.10E-02
IL6R	-1.060	transmembrane receptor	Inhibited	-2.623	6.17E-02
F7	-1.001	peptidase	Inhibited	-2.608	5.68E-02
INHBB	-1.126	growth factor	Inhibited	-2.578	3.62E-01
NFATC3	-1.006	transcription regulator	Inhibited	-2.577	6.17E-02
Pro-inflammatory Cytokine	NA	group	Inhibited	-2.564	3.36E-01
PAX3	-1.254	transcription regulator	Inhibited	-2.551	1.76E-02
TREM1	1.167	other	Inhibited	-2.547	6.77E-03
Vegf	NA	group	Inhibited	-2.547	3.36E-05
AKT1	1.072	kinase	Inhibited	-2.546	1.35E-04
ARNT2	-1.151	transcription regulator	Inhibited	-2.543	9.20E-05
CPE (includes EG:12876)	-1.137	peptidase	Inhibited	-2.543	1.57E-01
INSIG1	-1.154	other	Inhibited	-2.535	1.57E-01
IL31	-1.118	other	Inhibited	-2.531	3.97E-01
Mek	NA	group	Inhibited	-2.513	4.88E-03
USF2	-1.295	transcription regulator	Inhibited	-2.505	4.91E-02
PAX7	-1.096	transcription regulator	Inhibited	-2.505	4.10E-01
HOXC6	-1.044	transcription regulator	Inhibited	-2.502	6.26E-01
CYP51A1	1.298	enzyme	Inhibited	-2.496	3.01E-01
GABPA	1.105	transcription regulator	Inhibited	-2.495	7.50E-02
MITF	-1.077	transcription regulator	Inhibited	-2.469	2.15E-01
IL21	-1.200	cytokine	Inhibited	-2.465	2.33E-02
STAT3	-1.213	transcription regulator	Inhibited	-2.459	8.70E-09
GHRHR (includes EG:14602)	-1.037	G-protein coupled receptor	Inhibited	-2.449	3.01E-01
ERCC4	-1.081	enzyme	Inhibited	-2.449	5.23E-01
INSIG2	-1.076	other	Inhibited	-2.441	3.01E-01
CREB1	-1.003	transcription regulator	Inhibited	-2.437	2.15E-03
Growth hormone	NA	group	Inhibited	-2.436	4.24E-03
NDUFA13	1.021	enzyme	Inhibited	-2.434	1.00E00
GHSR (includes EG:100332193)	-1.022	G-protein coupled receptor	Inhibited	-2.433	5.74E-01
PI3	1.283	other	Inhibited	-2.425	5.74E-01
CLU	-1.988	other	Inhibited	-2.425	3.01E-01
IFNE	1.002	cytokine	Inhibited	-2.424	9.02E-02
ERG	-1.405	transcription regulator	Inhibited	-2.424	2.14E-03
GRN	-1.138	growth factor	Inhibited	-2.412	5.23E-01
GIP	1.073	other	Inhibited	-2.408	5.23E-01
HRH2	1.250	G-protein coupled receptor	Inhibited	-2.400	4.35E-01
NOTCH4	-1.088	transcription regulator	Inhibited	-2.398	5.50E-01
FOXO4	-1.201	transcription regulator	Inhibited	-2.385	3.26E-02
EP300	-1.010	transcription regulator	Inhibited	-2.382	3.91E-03
HDAC6	1.109	transcription regulator	Inhibited	-2.381	6.83E-02
PTK2 (includes EG:14083)	-2.350	kinase	Inhibited	-2.367	2.25E-02
AMPK	NA	complex	Inhibited	-2.364	8.11E-03
SMYD3	1.339	enzyme	Inhibited	-2.360	2.50E-01
HOXD3	-1.119	transcription regulator	Inhibited	-2.357	2.08E-01
STAT	NA	group	Inhibited	-2.347	5.68E-02
PCDH11X/PCDH11Y	1.262	other	Inhibited	-2.346	1.43E-01
VTN	-1.197	other	Inhibited	-2.336	9.02E-02
RUNX2	-1.941	transcription regulator	Inhibited	-2.310	3.24E-03
IL1	NA	group	Inhibited	-2.306	4.54E-03
AKT2	-1.083	kinase	Inhibited	-2.305	9.90E-02
NTRK2	-1.009	kinase	Inhibited	-2.300	1.30E-01
TGM2	1.119	enzyme	Inhibited	-2.293	4.05E-02
RBL1	1.349	other	Inhibited	-2.289	2.85E-02
MZF1	-1.028	transcription regulator	Inhibited	-2.287	3.97E-01

Supplementary Table 3 (con't). Predicted Upstream Regulator Molecules in CML Compared to Normal Stem Cells

Upstream Regulator*	Fold Change (Expression)†	Molecule Type	Predicted Activation State‡	Activation Z-score§	*P* of overlap||
NRAS	1.039	enzyme	Inhibited	-2.285	3.32E-02
MUC1	1.017	transcription regulator	Inhibited	-2.284	9.90E-02
SUZ12	1.083	enzyme	Inhibited	-2.277	1.26E-01
ITGAV	-1.134	other	Inhibited	-2.275	9.02E-02
NRG1 (includes EG:112400)	-1.060	growth factor	Inhibited	-2.271	1.60E-01
PAX2	-1.067	transcription regulator	Inhibited	-2.267	3.01E-01
RBPJ	-1.118	transcription regulator	Inhibited	-2.267	1.00E00
PLAUR	-2.537	transmembrane receptor	Inhibited	-2.262	2.61E-01
PRKACA	1.190	kinase	Inhibited	-2.249	2.97E-02
WNT3A	1.101	other	Inhibited	-2.237	1.72E-01
AGA	1.192	enzyme	Inhibited	-2.236	6.30E-01
RND3	-2.909	enzyme	Inhibited	-2.236	6.30E-01
FGFR1	-1.564	kinase	Inhibited	-2.228	4.55E-02
LTBP1	-1.370	other	Inhibited	-2.228	1.43E-01
LDB1	-1.018	transcription regulator	Inhibited	-2.219	4.35E-01
NPAS2	-1.097	transcription regulator	Inhibited	-2.219	5.23E-01
PP2A	NA	complex	Inhibited	-2.219	5.74E-01
BPI	1.024	transporter	Inhibited	-2.219	6.30E-01
PHOX2A	-1.142	transcription regulator	Inhibited	-2.219	6.30E-01
Akt	NA	group	Inhibited	-2.217	1.46E-04
GH1	-1.176	cytokine	Inhibited	-2.216	1.11E-03
ADAM15	1.078	peptidase	Inhibited	-2.213	5.74E-01
NR1I3	-1.060	ligand-dependent nuclear receptor	Inhibited	-2.207	2.88E-02
SIAH2	-1.031	transcription regulator	Inhibited	-2.207	5.23E-01
MIB1	-1.086	other	Inhibited	-2.203	1.00E00
Smad1/5/8-Smad4	NA	complex	Inhibited	-2.201	6.30E-01
Gcn5l	NA	group	Inhibited	-2.200	5.74E-01
ORAI1	1.173	ion channel	Inhibited	-2.200	6.30E-01
ITGA11	1.014	other	Inhibited	-2.200	6.30E-01
FOXD1	-1.043	transcription regulator	Inhibited	-2.197	4.77E-01
MTA1	1.042	transcription regulator	Inhibited	-2.195	1.50E-01
CDKN1A	-2.110	kinase	Inhibited	-2.191	1.36E-02
RXRG	-1.005	ligand-dependent nuclear receptor	Inhibited	-2.183	1.00E00
Fgfr	NA	group	Inhibited	-2.178	1.00E00
PAX9	-1.125	transcription regulator	Inhibited	-2.177	5.74E-01
ZBTB17	1.004	transcription regulator	Inhibited	-2.167	1.43E-01
TNFSF11	-1.027	cytokine	Inhibited	-2.166	3.41E-04
JUN	-1.322	transcription regulator	Inhibited	-2.162	1.61E-06
PTGER2	-1.595	G-protein coupled receptor	Inhibited	-2.160	7.50E-02
PPP3CA	1.075	phosphatase	Inhibited	-2.159	3.09E-02
CREBBP	-1.159	transcription regulator	Inhibited	-2.158	3.22E-02
NOS1	-1.069	enzyme	Inhibited	-2.158	3.97E-01
Pka catalytic subunit	NA	group	Inhibited	-2.158	1.00E00
ING4	1.281	transcription regulator	Inhibited	-2.157	4.77E-01
BAG1	1.229	other	Inhibited	-2.156	3.30E-01
LOXL2	-1.098	enzyme	Inhibited	-2.156	6.30E-01
PRKCH	-2.123	kinase	Inhibited	-2.147	5.74E-01
FOS	-2.113	transcription regulator	Inhibited	-2.144	8.74E-05
CLOCK	-1.060	transcription regulator	Inhibited	-2.144	3.64E-02
SLC30A3	-1.205	transporter	Inhibited	-2.138	4.77E-01
FLNA	-1.070	other	Inhibited	-2.138	1.00E00
STAT1/3/5 dimer	NA	complex	Inhibited	-2.137	7.13E-01
TRAF6	1.067	enzyme	Inhibited	-2.134	3.18E-01
KDM5B	1.002	transcription regulator	Inhibited	-2.130	2.61E-01
NR4A2	-2.930	ligand-dependent nuclear receptor	Inhibited	-2.129	3.54E-01
CAMK4	-1.136	kinase	Inhibited	-2.129	6.18E-01
TWIST2	NA	transcription regulator	Inhibited	-2.125	4.62E-01
IL9	-1.022	cytokine	Inhibited	-2.124	4.09E-01
SIM1 (includes EG:20464)	-1.005	transcription regulator	Inhibited	-2.112	6.25E-05
Collagen Alpha1	NA	group	Inhibited	-2.111	3.97E-01
ELF4	-1.148	transcription regulator	Inhibited	-2.109	5.68E-02
ADCYAP1 (includes EG:11516)	1.029	other	Inhibited	-2.065	4.58E-05
PTGER4	-1.666	G-protein coupled receptor	Inhibited	-2.063	1.19E-01
BMP7	1.058	growth factor	Inhibited	-2.048	8.53E-04
MEF2A	1.323	transcription regulator	Inhibited	-2.048	4.90E-01
GAST	1.055	other	Inhibited	-2.039	2.44E-02
TLR9	-1.042	transmembrane receptor	Inhibited	-2.038	6.54E-02
SMO	1.017	G-protein coupled receptor	Inhibited	-2.037	6.46E-01
TOB1	-1.598	transcription regulator	Inhibited	-2.036	2.47E-02
CD47	1.174	other	Inhibited	-2.035	3.30E-01
CCR1	-1.056	G-protein coupled receptor	Inhibited	-2.034	1.43E-01
STAT5A	1.018	transcription regulator	Inhibited	-2.034	2.17E-01
CYR61	-1.285	other	Inhibited	-2.033	5.18E-02
IFNG (includes EG:15978)	-1.056	cytokine	Inhibited	-2.031	4.06E-09
Retnlb	NA	other	Inhibited	-2.031	1.64E-02
CRY2	-1.235	enzyme	Inhibited	-2.029	3.97E-01

Supplementary Table 3 (con't). Predicted Upstream Regulator Molecules in CML Compared to Normal Stem Cells

Upstream Regulator*	Fold Change (Expression)†	Molecule Type	Predicted Activation State‡	Activation Z-score§	*P* of overlap‖
Fgf	NA	group	Inhibited	-2.028	8.22E-02
SELP	-4.856	other	Inhibited	-2.028	2.44E-01
NCOA6	1.261	transcription regulator	Inhibited	-2.026	2.74E-01
POMC	-1.066	other	Inhibited	-2.025	4.68E-02
PLAG1	-5.021	transcription regulator	Inhibited	-2.017	2.86E-01
ADAM12	-1.146	peptidase	Inhibited	-2.016	4.77E-01
NEUROD1	-1.066	transcription regulator	Inhibited	-2.011	7.50E-02
SRC (family)	NA	group	Inhibited	-2.006	3.61E-01
BLVRA	1.006	enzyme	Inhibited	-2.000	6.91E-01
NPC1L1 (includes EG:100329429)	-1.171	transporter	Inhibited	-2.000	6.91E-01
SLCO1C1	-1.055	transporter	Inhibited	-2.000	6.91E-01
CREB3L4	-1.070	transcription regulator	Inhibited	-2.000	6.91E-01
WDR5	NA	enzyme	Inhibited	-2.000	6.91E-01
Csf	NA	group	Inhibited	-2.000	6.91E-01
FGF13	1.117	growth factor	Inhibited	-2.000	6.91E-01
ITIH2	-1.087	other	Inhibited	-2.000	6.91E-01
FUT4	-1.168	enzyme	Inhibited	-2.000	6.91E-01
ITIH1	-1.077	other	Inhibited	-2.000	6.91E-01
LMX1A	1.118	transcription regulator	Inhibited	-2.000	6.91E-01
RBPJL	-1.081	transcription regulator	Inhibited	-2.000	1.00E00
MTF2 (includes EG:17765)	1.287	transcription regulator	Activated	2.000	6.30E-01
GCLM	-1.776	enzyme	Activated	2.000	6.30E-01
Tlr11	NA	other	Activated	2.000	6.91E-01
TLR10	-1.121	transmembrane receptor	Activated	2.000	6.91E-01
Tlr13	NA	other	Activated	2.000	6.91E-01
Tlr12	NA	other	Activated	2.000	6.91E-01
B-cell receptor	NA	complex	Activated	2.000	6.91E-01
SCAVENGER RECEPTOR CLASS A	NA	group	Activated	2.000	6.91E-01
PKIA	1.062	other	Activated	2.000	6.91E-01
Nppb	NA	other	Activated	2.000	6.91E-01
miR-339-5p (and other miRNAs w/seed CCCUGUC)	NA	mature microRNA	Activated	2.000	6.91E-01
miR-294-5p (and other miRNAs w/seed CUCAAAA)	NA	mature microRNA	Activated	2.000	6.91E-01
AMBN	-1.268	growth factor	Activated	2.000	6.91E-01
GPAM	1.390	enzyme	Activated	2.000	6.91E-01
PILRB	1.335	other	Activated	2.000	6.91E-01
PRKAR2B	1.123	kinase	Activated	2.005	4.35E-01
GABBR1	-1.020	G-protein coupled receptor	Activated	2.008	5.23E-01
EPHB6	1.004	kinase	Activated	2.047	2.28E-01
SOX2	NA	transcription regulator	Activated	2.059	4.68E-02
E2F1	1.096	transcription regulator	Activated	2.065	7.20E-06
SCAP	-1.005	other	Activated	2.076	6.65E-02
HAND1	1.002	transcription regulator	Activated	2.100	2.74E-01
NFKBIB	-1.037	transcription regulator	Activated	2.102	1.60E-01
ACTB	1.071	other	Activated	2.111	3.30E-01
IL4R	-1.357	transmembrane receptor	Activated	2.132	2.08E-01
ETV6	-1.170	transcription regulator	Activated	2.136	4.35E-01
Fibrinogen	NA	complex	Activated	2.155	1.89E-01
DES	-1.111	other	Activated	2.178	5.74E-01
miR-196a-5p (and other miRNAs w/seed AGGUAGU)	NA	mature microRNA	Activated	2.200	3.62E-01
APOB	-1.056	transporter	Activated	2.200	4.09E-01
SLAMF1	-1.228	transmembrane receptor	Activated	2.200	5.74E-01
TAF4 (includes EG:100149942)	1.137	transcription regulator	Activated	2.214	6.89E-02
miR-292-5p (and other miRNAs w/seed CUCAAAC)	NA	mature microRNA	Activated	2.214	1.00E00
miR-132-3p (and other miRNAs w/seed AACAGUC)	NA	mature microRNA	Activated	2.216	1.00E00
IRF5	-1.000	transcription regulator	Activated	2.222	1.50E-01
E2F2	1.109	transcription regulator	Activated	2.229	4.55E-02
miR-10a-5p (and other miRNAs w/seed ACCCUGU)	NA	mature microRNA	Activated	2.231	6.30E-01
POU2AF1	1.091	transcription regulator	Activated	2.261	1.00E00
CCL2	-1.311	cytokine	Activated	2.267	1.70E-02
KLF15	-1.057	transcription regulator	Activated	2.282	4.12E-01
HHEX	1.036	transcription regulator	Activated	2.298	3.97E-01
CR1L	NA	other	Activated	2.331	9.02E-02
PARP9	1.200	other	Activated	2.359	1.00E00
EHMT2	1.069	transcription regulator	Activated	2.375	2.28E-01
HNRNPK	-1.180	other	Activated	2.392	4.77E-01
PIK3CD	-1.130	kinase	Activated	2.401	6.46E-01
NOG	-1.254	other	Activated	2.416	1.39E-01
THY1	1.001	other	Activated	2.424	1.00E00
FHIT	NA	enzyme	Activated	2.436	3.85E-01
SMOC2	-1.090	other	Activated	2.449	5.74E-01
NRF1	-1.116	transcription regulator	Activated	2.470	3.36E-01
SMAD7	-1.504	transcription regulator	Activated	2.471	9.28E-04
GAB1	1.055	other	Activated	2.570	1.00E00
NPC1	1.085	transporter	Activated	2.573	3.57E-02
mir-34	NA	microRNA	Activated	2.592	3.62E-01
miR-222-3p (and other miRNAs w/seed GCUACAU)	NA	mature microRNA	Activated	2.607	1.31E-01

Supplementary Table 3 (con't). Predicted Upstream Regulator Molecules in CML Compared to Normal Stem Cells

Upstream Regulator*	Fold Change (Expression)†	Molecule Type	Predicted Activation State‡	Activation Z-score§	*P* of overlap||
CXCL2	-1.217	cytokine	Activated	2.622	3.62E-01
SPDEF	-1.044	transcription regulator	Activated	2.630	2.44E-02
S100A6	-1.260	transporter	Activated	2.641	2.98E-01
MYC	1.669	transcription regulator	Activated	2.646	5.35E-05
miR-92a-3p (and other miRNAs w/seed AUUGCAC)	NA	mature microRNA	Activated	2.701	1.89E-01
COBRA1	NA	other	Activated	2.714	7.13E-01
SATB1	-1.158	transcription regulator	Activated	2.716	6.59E-02
NME1 (includes EG:18102)	1.603	kinase	Activated	2.781	1.04E-01
CLCA2	-1.052	ion channel	Activated	2.896	3.30E-01
ZNF217	1.216	transcription regulator	Activated	2.967	1.10E-02
ARHGDIG	-1.115	other	Activated	3.000	1.00E00
FBN1	-1.294	other	Activated	3.220	2.28E-01
REST	-1.118	transcription regulator	Activated	3.291	9.81E-03
miR-19b-3p (and other miRNAs w/seed GUGCAAA)	NA	mature microRNA	Activated	3.413	3.30E-01
miR-17-5p (and other miRNAs w/seed AAAGUGC)	NA	mature microRNA	Activated	3.432	6.65E-02
CCND1	-1.396	other	Activated	3.683	3.40E-02
EP400	1.080	other	Activated	3.709	3.57E-02
Alpha catenin	NA	group	Activated	3.786	4.01E-03
TBX2	-1.016	transcription regulator	Activated	4.672	9.36E-03

Molecules in the Ingenuity® Knowledge Base (Ingenuity® Systems, www.ingenuity.com) with a p-value no greater than 0.05 or Z-score greater than 2 or less than -2 are represented in the table.

NA indicates not applicable.

*Indicates a molecule predicted to explain a significant portion of the expression differences between CML and normal stem cells, as determined by calculation of p-value and Z-score, as described (Ingenuity® Systems, www.ingenuity.com).

†Calculated between CML stem and normal stem cell populations from five CML or normal marrow donors from log2 transformed, default RMA background corrected array intensities. Positive values indicate upregulation of the upstream regulator in CML stem populations, and negative values indicate downregulation in CML stem cells compared to normal.

‡Activity of the molecule predicted by Z-score calculation. A Z-score greater than 2 is activated, and less than -2 is inhibited.

§Z-score is calculated as described (Ingenuity® Systems, www.ingenuity.com).

||P-value is calculated by Fisher's Exact test, as described (Ingenuity® Systems, www.ingenuity.com).

Supplementary Table 4. Alternatively Spliced Transcripts in CML Compared to Normal Stem Cells

Transcript Cluster ID*	Gene Symbol	RefSeq ID	Genomic Location†	*P* (Alternative Splicing)‡	*P* (Expression)§	Fold Change (Expression)||
2624385	CACNA1D	NM_000720	3p14.3	0.00E+00	5.70E-04	3.006
2858134	PDE4D	NM_001165899	5q12	2.79E-38	1.28E-02	1.382
2584018	DPP4	NM_001935	2q24.3	4.82E-32	2.39E-04	7.181
3323891	GAS2	NM_005256	11p14.3	9.88E-23	1.60E-06	15.444
3000984	ABCA13	NM_152701	7p12.3	3.79E-20	2.13E-02	-2.057
3198346	PTPRD	NM_002839	9p23-p24.3	2.84E-19	2.04E-04	4.848
3934111	SIK1	NM_173354	21q22.3	1.49E-18	1.15E-03	-2.347
3290875	ANK3	NM_020987	10q21	3.79E-18	7.02E-04	-2.764
2503374	GLI2	NM_005270	2q14	4.55E-18	4.88E-02	1.172
2749484	RXFP1	NM_021634	4q32.1	2.78E-16	6.63E-04	7.799
3484497	FRY	NM_023037	13q13.1	2.13E-15	3.68E-03	-2.989
2316379	SKI	NM_003036	1q22-q24	6.40E-14	3.69E-03	-1.576
3174121	MAMDC2	NM_153267	9q21.12	1.45E-13	7.91E-05	4.734
2880292	DPYSL3	NM_001197294	5q32	5.17E-13	4.89E-04	2.158
2926969	PDE7B	NM_018945	6q23-q24	5.41E-13	6.89E-03	1.778
2922215	MARCKS	NM_002356	6q22.2	5.67E-13	8.51E-07	2.196
2340529	PDE4B	NM_002600	1p31	2.77E-12	6.63E-03	-2.088
3491486	PCDH17	NM_001040429	13q21.1	3.17E-12	9.34E-03	-2.279
3850069	DNMT1	NM_001130823	19p13.2	2.16E-11	1.62E-02	1.493
2883440	ADAM19	NM_033274	5q33.3	4.55E-11	7.74E-03	1.443
2412799	ORC1	NM_004153	1p32	9.01E-11	5.74E-04	1.972
3252036	PLAU	NM_002658	10q24	1.10E-10	5.04E-03	1.722
3798778	FAM38B	NM_022068	18p11.22	2.00E-10	3.40E-05	13.515
2730746	SLC4A4	NM_001098484	4q21	2.98E-10	3.21E-03	2.584
2468811	ASAP2	NM_003887	2p25; 2p24	6.29E-10	1.01E-02	1.866
2333907	RNF220	NM_018150	1p34.1	6.52E-10	7.30E-01	1.026
3508696	N4BP2L2	NM_014887	13q13.1	9.38E-10	6.72E-01	-1.036
3415229	NR4A1	NM_002135	12q13	9.74E-10	3.45E-03	-2.148
2788926	NR3C2	NM_000901	4q31.1	1.40E-09	1.43E-02	-2.029
2340433	LEPR	NM_002303	1p31	1.74E-09	1.30E-02	2.265
2469213	KLF11	NM_003597	2p25	1.81E-09	6.77E-05	-2.237
2952834	KCNK5	NM_003740	6p21	2.11E-09	4.05E-04	2.244
3097152	MCM4	NM_005914	8q11.2	2.55E-09	5.02E-03	2.247
2569215	ST6GAL2	NM_032528	2q11.2-q	2.87E-09	3.33E-03	-2.258
3544525	FOS	NM_005252	14q24.3	3.70E-09	1.13E-02	-2.025
3479181	POLE	NM_006231	12q24.3	4.42E-09	2.46E-03	1.762
2987632	TTYH3	NM_025250	7p22	5.06E-09	2.56E-02	1.199
3613300	NIPA2	NM_001184889	15q11.2	6.68E-09	3.54E-02	1.323
3242353	CREM	NM_183013	10p11.21	8.54E-09	7.72E-03	-1.870
3070309	CADPS2	NM_017954	7q31.3	1.06E-08	2.56E-03	-2.023
2588127	ATP5G3	NM_001190329	2q31.1	1.93E-08	7.88E-03	1.335
3911217	PMEPA1	NM_020182	20q13.31-q13.33	2.12E-08	2.82E-02	-1.665
2897899	SOX4	NM_003107	6p22.3	2.54E-08	3.84E-02	1.243
2560195	PCGF1	NM_032673	2p13.1	2.63E-08	2.14E-02	1.269
2319252	SPSB1	NM_025106	1p36.22	2.77E-08	3.21E-02	-1.950
3579546	WARS	NM_004184	14q32.31	3.95E-08	2.79E-01	1.165
3606304	AKAP13	NM_006738	15q24-q25	5.04E-08	7.23E-03	-1.352
2736322	PDLIM5	NM_006457	4q22	6.27E-08	9.01E-01	1.014
2508520	KYNU	NM_003937	2q22.2	6.50E-08	1.13E-03	3.525
2515050	GORASP2	NM_015530	2q31.1-q31.2	7.43E-08	3.37E-03	1.337
3411721	CNTN1	NM_001843	12q11-q12	7.90E-08	2.88E-03	-4.067
2776372	WDFY3	NM_014991	4q21.23	9.69E-08	1.66E-03	-1.889
3610804	IGF1R	NM_000875	15q26.3	9.71E-08	2.17E-03	-2.499
2394626	ACOT7	NM_007274	1p36	1.12E-07	1.01E-01	1.132
3802602	CDH2	NM_001792	18q11.2	1.28E-07	3.33E-04	-4.658
3279982	PTPLA	NM_014241	10p14-p13	1.34E-07	1.62E-03	1.583
4021149	SMARCA1	NM_003069	Xq25	1.34E-07	8.01E-03	2.846
2455418	PTPN14	NM_005401	1q32.2	1.52E-07	2.02E-02	2.834

Supplementary Table 4 (con't). Alternatively Spliced Transcripts in CML Compared to Normal Stem Cells

Transcript Cluster ID*	Gene Symbol	RefSeq ID	Genomic Location†	*P* (Alternative Splicing)‡	*P* (Expression)§	Fold Change (Expression)||
2905169	CDKN1A	NM_000389	6p21.2	1.52E-07	7.75E-03	-2.138
3415193	GRASP	NM_181711	12q13.13	1.58E-07	6.94E-03	-1.650
3525234	IRS2	NM_003749	13q34	1.95E-07	1.08E-03	-1.368
2447414	NCF2	NM_000433	1q25	2.62E-07	8.18E-04	1.621
3944690	CYTH4	NM_013385	22q12.3-q13.1	3.34E-07	2.60E-03	1.478
2520429	MYO1B	NM_001130158	2q12-q34	3.56E-07	1.38E-02	3.013
3585905	APBA2	NM_005503	15q11-q12	3.71E-07	5.26E-03	1.561
2475628	YPEL5	NM_001127401	2p23.1	3.94E-07	5.61E-04	-1.795
3673684	CDT1	NM_030928	16q24.3	4.17E-07	2.82E-03	1.274
3203482	BAG1	NM_004323	9p12	4.55E-07	2.27E-03	1.168
3741547	P2RX5	NM_002561	17p13.3	4.99E-07	4.13E-04	1.494
2748198	KIAA0922	NM_001131007	4q31.3	5.05E-07	2.06E-04	1.790
3367788	DCDC5	NM_020869	11p14.1	5.36E-07	4.63E-02	1.712
3012381	AKAP9	NM_005751	7q21-q22	5.39E-07	2.39E-01	1.183
3555817	ZNF219	NM_016423	14q11	5.88E-07	7.95E-02	-1.161
2564599	MRPS5	NM_031902	2p11.2-q11.2	6.20E-07	2.47E-03	1.323
3720921	RARA	NM_000964	17q21	6.29E-07	1.24E-03	-1.975
2513471	SCN2A	NM_021007	2q24.3	8.18E-07	9.91E-03	2.112
3320717	MICAL2	NM_014632	11p15.3	8.34E-07	2.29E-02	1.496
3976062	UBA1	NM_003334	Xp11.23	8.90E-07	8.48E-01	-1.019
2732391	CCNG2	NM_004354	4q21.1	8.99E-07	3.27E-01	1.114
2733287	PRDM8	NM_020226	4q21	9.09E-07	5.29E-02	1.282
2971801	MAN1A1	NM_005907	6q22	9.10E-07	1.52E-02	-2.163
3970642	CDKL5	NM_001037343	Xp22	9.40E-07	3.05E-02	-1.519
2658785	FAM43A	NM_153690	3q29	1.02E-06	1.35E-02	-1.412
2470838	MYCN	NM_005378	2p24.3	1.11E-06	3.56E-04	1.885
3507282	FLT1	NM_002019	13q12	1.13E-06	1.45E-02	-1.424
2877257	BRD8	NM_139199	5q31	1.17E-06	1.59E-04	1.483
2709778	BCL6	NM_001706	3q27	1.26E-06	2.45E-03	-2.264
3249587	SIRT1	NM_012238	10q21.3	1.33E-06	1.51E-02	-1.425
2620448	CLEC3B	NM_003278	3p22-p21.3	1.36E-06	1.48E-02	-2.136
2343334	GIPC2	NM_017655	1p31.1	1.52E-06	5.28E-03	-3.367
3181976	NR4A3	NM_006981	9q22	1.55E-06	6.18E-03	-2.725
2647647	TSC22D2	NM_014779	3q25.1	1.76E-06	3.16E-03	-1.797
3866898	LIG1	NM_000234	19q13.2-q13.3	1.79E-06	1.81E-03	1.313
2437152	THBS3	NM_007112	1q21	1.80E-06	2.71E-01	1.154
2882098	SPARC	NM_003118	5q31.3-q32	1.81E-06	4.47E-02	1.773
2674047	LAMB2	NM_002292	3p21	1.82E-06	1.40E-02	1.246
3648995	ERCC4	NM_005236	16p13.12	1.87E-06	9.37E-01	-1.015
2686458	ABI3BP	NM_015429	3q12	2.11E-06	3.31E-02	-2.633
3385769	CTSC	NM_001814	11q14.2	2.33E-06	2.24E-03	1.785
2694314	GATA2	NM_032638	3q21.3	2.36E-06	5.32E-02	1.223
3969358	EGFL6	NM_015507	Xp22	2.41E-06	6.35E-02	2.080
2680046	ADAMTS9	NM_182920	3p14.1	2.49E-06	2.61E-02	-1.291
2403740	SRSF4	NM_005626	1p35.3	2.72E-06	1.61E-02	1.445
2783788	PRDM5	NM_018699	4q25-q26	2.97E-06	3.95E-02	-1.700
3237396	CACNB2	NM_201571	10p12	3.29E-06	1.36E-02	2.487
2628682	ARL6IP5	NM_006407	3p14	3.32E-06	2.83E-02	1.454
3587495	SCG5	NM_001144757	15q13-q14	3.34E-06	3.44E-03	-1.916
3662774	GPR114	NM_153837	16q21	3.43E-06	1.11E-04	1.282
3865119	ZNF296	NM_145288	19q13.32	3.63E-06	2.18E-01	-1.107
3457824	TIMELESS	NM_003920	12q12-q13	4.31E-06	1.21E-02	1.740
2334052	C1orf228	NM_001145636	1p34.1	4.34E-06	1.48E-01	1.240
3798829	FAM38B	NM_022068	18p11.22	4.60E-06	1.77E-04	8.156
3724591	C17orf57	NM_152347	17q21.32	4.66E-06	9.37E-02	-2.163
2400793	HSPG2	NM_005529	1p36.1-p34	4.72E-06	3.87E-01	1.064
2841491	C5orf41	NM_153607	5q35.1	4.76E-06	3.09E-01	1.167
3066297	SRPK2	NM_182691	7q22-q31.1	5.13E-06	8.92E-01	1.016

Supplementary Table 4 (con't). Alternatively Spliced Transcripts in CML Compared to Normal Stem Cells

Transcript Cluster ID*	Gene Symbol	RefSeq ID	Genomic Location†	*P* (Alternative Splicing)‡	*P* (Expression)§	Fold Change (Expression)||
3778504	RAB31	NM_006868	18p11.3	5.23E-06	2.86E-04	4.647
2582124	NR4A2	NM_006186	2q22-q23	5.25E-06	7.91E-03	-2.797
2778440	UNC5C	NM_003728	4q21-q23	5.29E-06	1.08E-02	1.285
2408041	HPCAL4	NM_016257	1p34.2	5.54E-06	2.07E-02	-1.692
2580802	RND3	NM_005168	2q23.3	5.73E-06	1.31E-03	-2.296
2363902	DUSP12	NM_007240	1q21-q22	5.99E-06	1.04E-02	1.368
3807261	SMAD7	NM_005904	18q21.1	6.20E-06	1.84E-02	-1.678
3473436	TESC	NR_031766	12q24.22	6.38E-06	1.27E-02	1.737
2521574	PLCL1	NM_006226	2q33	6.57E-06	4.18E-04	-3.670
3633403	SIN3A	NM_015477	15q24.2	6.76E-06	8.87E-01	1.015
3417249	ERBB3	NM_001982	12q13	6.86E-06	8.74E-01	-1.012
3914021	GMEB2	NM_012384	20q13.33	7.13E-06	2.19E-01	-1.159
2509988	LYPD6B	NM_177964	2q23.2	7.47E-06	4.01E-03	-1.665
2522247	AOX1	NM_001159	2q33	7.52E-06	8.10E-03	-1.507
2473936	KCNK3	NM_002246	2p23	9.46E-06	1.86E-02	-1.201
3485292	NBEA	NM_015678	13q13	9.66E-06	4.37E-04	-1.938
2358426	ADAMTSL4	AK023606	1q21.3	9.87E-06	4.14E-02	-1.700
3352948	SORL1	NM_003105	11q23.2-q24.2	9.89E-06	9.59E-04	-2.154
3577683	SERPINA9	NM_175739	14q32.13	1.06E-05	6.35E-01	1.059
3772158	TK1	NM_003258	17q23.2-q25.3	1.07E-05	1.30E-04	1.343
2364016	NOS1AP	NM_014697	1q23.3	1.09E-05	8.06E-01	1.021
3887302	CD40	NM_001250	20q12-q13.2	1.10E-05	1.76E-02	-1.522
3881391	ID1	NM_181353	20q11	1.13E-05	1.57E-03	-2.084
3504526	LATS2	NM_014572	13q11-q12	1.14E-05	9.21E-03	-1.298
3815233	ELANE	NM_001972	19p13.3	1.16E-05	4.96E-03	-1.421
2623515	ALAS1	NM_000688	3p21.1	1.16E-05	3.65E-02	1.447
2976360	PERP	NM_022121	6q24	1.17E-05	1.85E-03	-3.289
3536434	SAMD4A	NM_015589	14q22.2	1.18E-05	1.24E-01	-1.167
3716151	ANKRD13B	NM_152345	17q11.2	1.20E-05	1.23E-01	1.194
3772279	SOCS3	NM_003955	17q25.3	1.36E-05	6.52E-04	-1.765
3228373	TSC1	NM_000368	9q34	1.40E-05	2.02E-01	1.159
2601287	AP1S3	NM_001039569	2q36.1	1.46E-05	2.56E-04	1.495
2652801	NLGN1	NM_014932	3q26.31	1.55E-05	6.71E-03	-2.651
2905469	RNF8	NM_003958	6p21.3	1.59E-05	1.36E-02	1.464
2395890	CLSTN1	NM_001009566	1p36.22	1.60E-05	5.37E-01	-1.040
3754227	MYO19	NM_001163735	17q12	1.61E-05	1.61E-02	1.305
3261492	NOLC1	NM_004741	10q24.32	1.64E-05	1.49E-01	1.305
4027176	FLNA	NM_001456	Xq28	1.73E-05	3.71E-01	-1.080
3319937	WEE1	NM_003390	11p15.3-p15.1	1.76E-05	3.38E-03	1.504
3517251	DACH1	NM_080759	13q22	1.76E-05	4.75E-03	-2.013
3545130	VASH1	NM_014909	14q24.3	1.81E-05	1.26E-01	-1.216
3859761	DMKN	NM_001126056	19q13.12	1.85E-05	1.93E-02	-1.361
3144934	GEM	NM_005261	8q13-q21	1.86E-05	6.15E-04	-2.653
3895118	CPXM1	NM_019609	20p13-p12.3	1.93E-05	2.72E-03	1.632
2931391	MTHFD1L	NM_015440	6q25.1	1.99E-05	4.74E-01	-1.105
3449068	TMTC1	NM_001193451	12p11.22	2.22E-05	4.60E-02	-1.695
3815649	CIRBP	NR_023312	19p13.3	2.24E-05	1.07E-01	-1.192
2332812	ERMAP	NM_001017922	1p34.2	2.38E-05	1.05E-02	1.205
4017798	KCNE1L	NM_012282	Xq22.3	2.49E-05	1.60E-02	-1.258
2475209	PPP1CB	NM_002709	2p23	2.66E-05	1.21E-03	-1.509
3868753	KLK5	NM_012427	19q13.33	2.72E-05	3.23E-01	1.051
2665720	ZNF385D	NM_024697	3p24.3	2.95E-05	6.44E-02	-2.627
3703885	SLC7A5	NM_003486	16q24.3	3.03E-05	3.59E-02	-1.830
2650199	SMC4	NM_005496	3q26.1	3.04E-05	1.52E-02	1.416
2891341	IRF4	NM_002460	6p25-p23	3.22E-05	1.70E-02	-1.634
2633691	TMEM45A	NM_018004	3q12.2	3.34E-05	4.43E-03	1.947
2832052	HARS2	NM_012208	5q31.3	3.44E-05	7.13E-04	1.549
2316953	PRDM16	NM_022114	1p36.23-p33	3.49E-05	9.98E-03	-1.491

Supplementary Table 4 (con't). Alternatively Spliced Transcripts in CML Compared to Normal Stem Cells

Transcript Cluster ID*	Gene Symbol	RefSeq ID	Genomic Location†	*P* (Alternative Splicing)‡	*P* (Expression)§	Fold Change (Expression)||
3254337	C10orf57	NM_025125	10q22.3	3.52E-05	1.01E-01	1.592
3888613	CEBPB	NM_005194	20q13.1	3.53E-05	2.88E-01	-1.105
3904119	CPNE1	NM_152930	20q11.22	3.57E-05	5.58E-01	1.060
3932131	PSMG1	NM_003720	21q22.3	3.72E-05	4.17E-03	1.792
2548617	CDC42EP3	NM_006449	2p21	3.76E-05	9.88E-02	1.373
2796510	MLF1IP	NM_024629	4q35.1	3.88E-05	2.29E-02	2.400
3779579	TUBB6	NM_032525	18p11.21	3.89E-05	1.24E-02	1.603
2484970	EHBP1	NM_015252	2p15	3.96E-05	6.92E-02	-1.361
3226592	WDR34	NM_052844	9q34.11	3.98E-05	1.28E-02	1.084
3032647	DPP6	NM_001039350	7q36.2	4.27E-05	3.08E-01	-1.109
2502762	STEAP3	NM_182915	2q14.2	4.47E-05	8.73E-02	1.273
2644014	PCCB	NM_001178014	3q21-q22	4.62E-05	2.73E-02	1.312
2725061	LIMCH1	NM_014988	4p13	5.26E-05	8.59E-03	-1.880
3775842	TYMS	NM_001071	18p11.32	5.42E-05	5.90E-03	2.090
3693788	SLC38A7	NM_018231	16q21	5.53E-05	4.96E-01	1.049
7385641	CLSTN2	NM_022131	3q23	5.60E-05	3.09E-01	-1.111
3877221	C20orf7	NR_029377	20p12.1	5.70E-05	2.85E-02	1.348
2646125	CHST2	NM_004267	3q24	6.02E-05	8.06E-01	-1.015
2878273	HBEGF	NM_001945	5q23	6.11E-05	3.38E-02	-1.389
3833992	CYP2S1	NM_030622	19q13.1	6.22E-05	3.67E-02	1.221
3897505	JAG1	NM_000214	20p12.1-p11.23	6.62E-05	6.18E-05	-1.904
3438061	GPR133	NM_198827	12q24.33	6.68E-05	7.06E-01	1.035
2475678	LBH	NM_030915	2p23.1	7.04E-05	1.46E-02	-1.680
3241601	C10orf68	NM_024688	10p11.22	7.13E-05	7.00E-02	-1.394
3839718	CD33	NM_001772	19q13.3	7.45E-05	1.43E-03	1.796
3267382	INPP5F	NM_014937	10q26.11	7.67E-05	9.67E-01	-1.003
3675285	WDR24	NM_032259	16p13.3	7.85E-05	9.93E-01	-1.001
3257098	FAS	NM_000043	10q24.1	7.85E-05	2.19E-02	1.983
3836432	QPCTL	NM_017659	19q13.32	7.87E-05	8.78E-01	1.009
2346074	ZNF326	NM_182976	1p22.2	7.87E-05	3.51E-01	1.130
3027808	AGK	NM_018238	7q34	8.02E-05	8.05E-01	-1.039
3932148	BRWD1	NM_033656	21q22.2	8.26E-05	1.95E-01	-1.136
3922793	PDE9A	NM_002606	21q22.3	8.36E-05	1.62E-02	1.255
2735027	SPP1	NM_001040058	4q22.1	9.08E-05	2.14E-02	-3.203
3127978	NKX3-1	NM_006167	8p21	9.11E-05	2.62E-02	-1.377
3424174	MYF5	NM_005593	12q21	9.41E-05	5.98E-01	1.063
2883878	EBF1	NM_024007	5q34	9.60E-05	1.05E-03	2.282
3129065	CLU	NM_001831	8p21-p12	9.65E-05	1.58E-02	-1.705
3482572	WASF3	NM_006646	13q12	9.66E-05	2.43E-02	-1.990
2571457	CKAP2L	NM_152515	2q13	9.86E-05	1.99E-02	1.648
2893895	BMP6	NM_001718	6p24-p23	1.01E-04	4.68E-02	-2.824
2602304	TM4SF20	NM_024795	2q36.3	1.02E-04	6.69E-01	-1.063
2577856	LCT	NM_002299	2q21	1.04E-04	1.05E-01	1.219
2882196	ATOX1	NM_004045	5q32	1.04E-04	1.30E-03	1.613
2676927	SELK	NM_021237	3p21.31	1.10E-04	1.71E-01	-1.137
3188111	PTGS1	NM_000962	9q32-q33.3	1.11E-04	1.17E-02	-2.468
2496727	MAP4K4	NM_145686	2q11.2-q12	1.12E-04	1.97E-02	1.529
3669059	GABARAPL2	NM_007285	16q22.1	1.12E-04	1.63E-02	-1.534
2544201	TP53I3	NM_004881	2p23.3	1.13E-04	5.94E-04	1.414
3219215	KLF4	NM_004235	9q31	1.14E-04	9.00E-04	-1.730
2577958	DARS	NM_001349	2q21.3	1.16E-04	9.95E-01	-1.001
3715874	ERAL1	NM_005702	17q11.2	1.16E-04	4.16E-04	1.626
3359529	CARS	NR_036542	11p15.5	1.17E-04	2.15E-01	1.222
2925841	ARG1	NM_000045	6q23	1.18E-04	5.03E-01	-1.080
4017381	TSC22D3	NM_198057	Xq22.3	1.19E-04	4.07E-02	-1.672
3645947	CLUAP1	NM_015041	16p13.3	1.19E-04	2.39E-02	1.702
3236958	VIM	NM_003380	10p13	1.20E-04	1.78E-01	-1.164
2474977	FOSL2	NM_005253	2p23.3	1.20E-04	3.80E-03	-2.081

Supplementary Table 4 (con't). Alternatively Spliced Transcripts in CML Compared to Normal Stem Cells

Transcript Cluster ID*	Gene Symbol	RefSeq ID	Genomic Location†	*P* (Alternative Splicing)‡	*P* (Expression)§	Fold Change (Expression)||
2359885	SLC27A3	NM_024330	1q21.3	1.25E-04	4.76E-03	1.289
2656971	RTP1	NM_153708	3q27.3	1.28E-04	6.73E-01	1.052
2341083	GADD45A	NM_001924	1p31.2	1.28E-04	4.40E-03	-1.613
3453837	TUBA1A	NM_006009	12q13.12	1.29E-04	2.31E-02	-1.402

Genes with alternative transcript p-value < 0.01 are represented in the table. All calculations were made between CML stem and normal stem cell populations from five CML or normal marrow donors from log2 transformed, default RMA background corrected array intensities.

*From Human Exon 1.0 ST whole genome gene expression microarrays (Affymetrix, Santa Clara, CA).

†Genomic coordinates refer to the human reference genome hg19 (GRCh37).

‡Alternative splicing p-value resulting from ANOVA analysis of exon-level microarray data.

§Expression p-value resulting from ANOVA analysis of gene-level microarray data.

||Positive values indicate upregulation of the upstream regulator in CML stem populations, and negative values indicate downregulation in CML stem cells compared to normal.
